# DnaC traps DnaB as an open ring and remodels the domain that binds primase

**DOI:** 10.1093/nar/gkv961

**Published:** 2015-09-29

**Authors:** Sundari Chodavarapu, A. Daniel Jones, Michael Feig, Jon M. Kaguni

**Affiliations:** 1 Department of Biochemistry and Molecular Biology, Michigan State University, East Lansing, MI 48824-1319, USA; 2 Department of Chemistry, Michigan State University, East Lansing, MI 48824–1319, USA

## Abstract

Helicase loading at a DNA replication origin often requires the dynamic interactions between the DNA helicase and an accessory protein. In *E. coli*, the DNA helicase is DnaB and DnaC is its loading partner. We used the method of hydrogen/deuterium exchange mass spectrometry to address the importance of DnaB–DnaC complex formation as a prerequisite for helicase loading. Our results show that the DnaB ring opens and closes, and that specific amino acids near the N-terminus of DnaC interact with a site in DnaB's C-terminal domain to trap it as an open ring. This event correlates with conformational changes of the RecA fold of DnaB that is involved in nucleotide binding, and of the AAA+ domain of DnaC. DnaC also causes an alteration of the helical hairpins in the N-terminal domain of DnaB, presumably occluding this region from interacting with primase. Hence, DnaC controls the access of DnaB by primase.

## INTRODUCTION

In free-living organisms, DNA replication is a cell-cycle regulated event that depends on the coordinated activities of a macromolecular machine named the replisome, whose components include a primase that synthesizes primers extended by the DNA polymerase as it duplicates chromosomes, and a DNA helicase located at the replication fork where it unwinds the parental duplex DNA (reviewed in (1)). These components interact dynamically. As an example, for primase of *E. coli* to form primers, it must interact with the DNA helicase named DnaB bound to the parental DNA strand that serves as the lagging strand template (2,3,4). After primer formation, the mechanism of primer transfer from DnaB-associated primase to DNA polymerase III holoenzyme apparently requires an interaction of the χ subunit of the polymerase with SSB in contact with primase, leading to displacement of primase from the primer (5). Primase then synthesizes another primer when it subsequently binds to DnaB now located at a distal DNA site by virtue of the moving replication fork. DnaC also interacts with DnaB to form the DnaB–DnaC (BC) complex, but dissociates from DnaB after the helicase has loaded at the *E. coli* replication origin (*oriC*) (6). Hence, conformational changes in both DnaB and DnaC must take place for DNA replication to occur.

Bacterial and archaeal replicative helicases are barrel-shaped toroids composed of six identical subunits (4,7). Their eukaryotic counterparts are also toroids but of non-identical subunits (7). Several X-ray crystallographic structures of bacterial DnaB have been described, one of which is a closed ring (8–11). Another bound to single-stranded DNA and GDP–AlF_4_, an analogue of GTP, is an open ring analogous to a lock washer with a right-handed turn in which the DNA is in the interior channel (12). The opening between adjacent protomers of DnaB enables entry of single-stranded DNA to the interior of DnaB (13). For unwinding of duplex DNA, these helicases translocate on one of the two strands of the parental DNA that passes through the central cavity, and hydrolyze ATP or other nucleotides during unwinding (14–18). A recent model of *Geobacillus stearothermophilus* DnaB in a complex with single-stranded DNA and an ATP analogue proposes substantial sequential movement of the subunits along single-stranded DNA during unwinding (12). These enzymes and those of some bacteriophage (gene 4 helicase of bacteriophage T7, and G4OP helicase of bacteriophage SPP1) share a common subunit structure of an N-terminal domain (NTD) joined to a larger C-terminal domain (CTD) via an α-helix linker (13,19–20). For DnaB, the NTD of each protomer interacts pairwise with the neighboring NTD to form a trimer of dimers (8,21). The CTD is structurally similar to the ATPase domain of RecA, and functions to coordinate nucleotide hydrolysis with mechanical work in the form of DNA unwinding (22). In the X-ray crystallographic structure of the closed DnaB ring, the CTDs are arranged with C6 symmetry (8–10).

Under physiological conditions, the binding of *E. coli* DnaB to DNA occurs initially at *oriC* (before its movement as part of the replisome), and requires that the helicase is complexed to DnaC in which a maximum of six DnaC monomers are bound (23–25). A model of an open-ring structure has been proposed (see below). Although DnaC binds with comparable affinities to ATP and ADP (26,27), BC complex formation does not require ATP. In contrast, ATP binding by DnaC is needed for helicase loading at *oriC* (6,27), which correlates with studies of the AAA+ domain of *A. aeolicus* DnaC that suggest that ATP promotes the formation of a spiral DnaC oligomer (28). The ability of DnaB and single-stranded DNA to stimulate the feeble ATPase activity of DnaC suggests that the loading of DnaB onto the unwound region of *oriC* leads to the hydrolysis of ATP bound to DnaC and the subsequent dissociation of DnaC from DnaB (6,27–29).

Early cryo-electron microscopic (cryo-EM) analysis of the BC complex (21) and fluorescence energy transfer experiments (23) strongly suggest that DnaC interacts with the CTD of DnaB. More recently, X-ray crystallography of *G. stearotheromophilus* DnaB complexed to *B. subtilis* DnaI, which like *E. coli* DnaC forms a complex with its respective DNA helicase (30), shows that DnaI is localized at the CTD of DnaB (31). Reconstruction of the *E. coli* BC complex by merging cryo-EM data with X-ray crystallographic structures of DnaB and the AAA+ domain of DnaC from thermophilic bacteria also place it at the CTD of DnaB (32). This model of an open ring structure, which incorporates a form of DnaC lacking its N-terminal region that is known to interact with DnaB (33), suggests that the binding of DnaC induces a conformational change in DnaB from a closed to an open ring. Despite these studies, the sites in DnaB that make contact with DnaC in the BC complex are unknown, which is essential to understand the role of DnaC in modulating the structure and function of DnaB. We sought to understand protein dynamics in assembly of the complex, and to discern sites of interaction between DnaB and DnaC by integrating the structural information of the static structures summarized above with the results obtained from hydrogen/deuterium exchange (HDX) experiments. The results herein describe that the interface separating each CTD in the DnaB ring naturally opens, and that the binding of DnaC captures DnaB in the open ring state. DnaC bound to DnaB also causes occlusion of a domain in the NTD of DnaB that acts to bind primase.

## MATERIALS AND METHODS

Pepsin, D_2_O (99.9 atom% D) and ATP were purchased from Sigma–Aldrich Chemical Co. ATPγS and Poros-20AL were from EMD-Millipore and Lifetechnologies, respectively. All other chemicals were HPLC grade. DnaB and DnaC were purified from overproducing strains essentially as described (6,34). The BC complex was assembled by incubation of hexameric DnaB with a 2-fold molar excess of DnaC at 20°C in Buffer A (25 mM HEPES-KOH pH 7.6, 5 mM MgCl_2_, 10% (v/v) glycerol, 20 mM NaCl, 2 mM DTT) and 2 mM ATP or 10 μM ATPγS followed by isolation on a precalibrated Superose 12 column equilibrated in the respective buffer. Quantitative densitometry of peak fractions visualized in a Coomassie Blue-stained SDS-polyacrylamide gel confirmed the composition of the DnaB_6_–DnaC_6_ complex. Given the binding affinities of DnaB (K_D_ = 2.8 μM ATP, 0.1 μM ATPγS; (35)) and DnaC (K_D_ ∼ 6–13 μM ATP, K_I_ = 6–12 μM ATPγS in competition assays with ATP; (25–27)), these nucleotide concentrations were chosen on the basis that both proteins should be bound to ATP or ATPγS. Similar HDX results were obtained with buffer containing 10 μM and 100 μM ATPγS, but only the data with the lower concentration is shown.

A column (2 mm x 50 mm) containing pepsin immobilized on Poros-20AL resin was prepared essentially as described (36). After its equilibration in 0.099% (v/v) formic acid, samples (100 μl) prepared as described below for online pepsin digestion were applied to the column using an external injection port at 0.1 ml/min for 1 min with an external Shimadzu LC-20AD pump. At this and subsequent steps, the valves, columns, injection port and solutions were maintained on ice. After stopping the pump for 2 min, the peptic fragments were trapped and desalted on an Acquity UPLC BEH C18 1.7 μm VanGuard column (Waters) by running the external pump at 0.5 ml/min. The peptides were then eluted by valve switching of liquid flow, using a Waters 2777 autosampler, to load onto to an Ascentis Express Peptide ES-C18 analytical column (5 cm x 2.1 mm; Supelco) and the column was washed with 0.099% formic acid plus 1% acetonitrile for 1 min at a flow rate of 0.3 ml/min. Analytical liquid flows were provided by a Waters Acquity Binary Solvent Manager. The peptides were then separated by elution at the same flow rate with a gradient from 0.099% formic acid plus 1% acetonitrile to 0.07% formic acid and 30% acetonitrile for 3 min, followed by a gradient from the latter solution to 0.001% formic acid and 99% acetonitrile for 3 min, and then washed in this solution for 1 min to end the procedure. During chromatography, the eluant was directed to a Xevo G2S QTof mass spectrometer (Waters) operated using electrospray ionization in positive-ion mode. Mass spectra were acquired in continuum mode over *m/z* 50–2000 at a mass resolution (M/ΔM) of approximately 20 000 (full width-half maximum). Data analysis was performed using HX Express software and MS tools (37,38).

As a control and also to determine the identities of undeuterated peptides, DnaB or DnaC (3 μg each, respectively) in 10 μl of Buffer A supplemented with 2 mM ATP was diluted 10-fold at 20°C with this buffer. After the addition of an equal volume of ice-cold 1% formic acid, online pepsin digestion and analysis were performed in MS/MS mode as described above. Using Biolynx software (Waters), the sequence information obtained from the analysis of peptides was compared with the amino acid sequence of DnaB and DnaC. As another control to measure the level of back-exchange during pepsin treatment, the fully deuterated proteins were analyzed. Depending on the individual peptide, back-exchange was from 30–50%, which is similar to studies by others (39). Briefly, the proteins (3 μg each) were diluted 10-fold into the above buffer but made with D_2_O, and incubated for 24 h at 20°C before the addition of formic acid, online pepsin digestion and analysis by mass spectrometry. To measure H/D exchange at various time intervals, DnaB, DnaC or the BC complex (3 μg each, respectively) in 10 μl of Buffer A supplemented with ATP or ATPγS was diluted with this buffer but made with D_2_O. At the indicated times, the reactions were placed on ice, and H/D exchange was quenched by promptly adding an equal volume of chilled 1% formic acid. Samples were then immediately injected onto the pepsin column followed by mass spectrometry analysis as described above. To determine the reproducibility of measurements, at least three independent sets of experiments with ATP or two with ATPγS were performed from which the standard deviation or range was calculated.

Deuteration percentages were calculated using the following formula:% D = 100(M_pd_−M_nd_)/N) where M_pd_ is the mass of the partially deuterated peptide, M_nd_ is the mass of the non-deuterated peptide, and N is the total number of exchangeable amide hydrogens within the peptide.

## RESULTS

**Figure 1.**
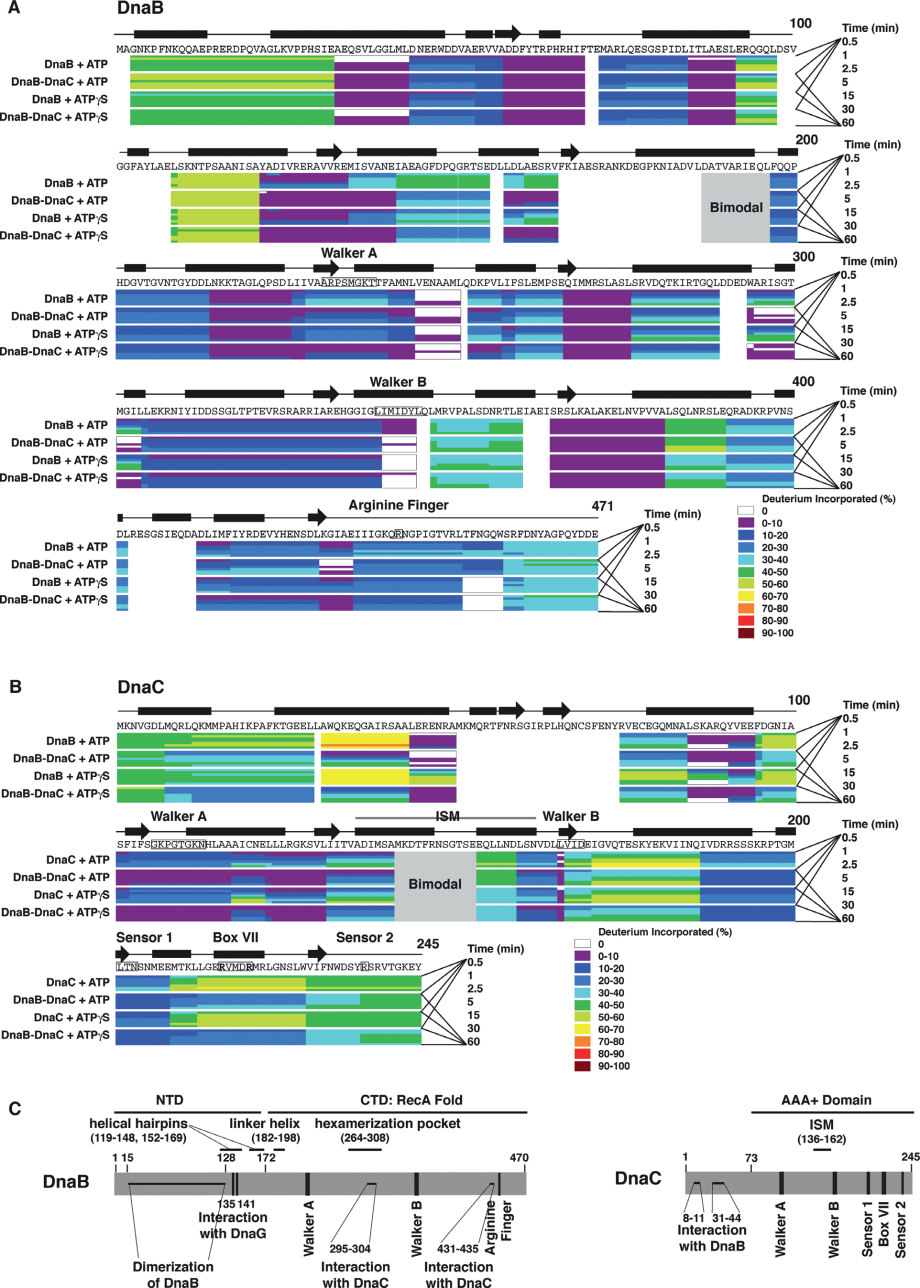
H/D exchange by peptides of DnaB and DnaC, color coded by percentage of deuterium exchanged. Deuteration of respective peptides of DnaB (panel **A**), DnaC (panel **B**) or the BC complex (panel **A**, **B**) in the presence of ATP or ATPγS was measured at the times indicated, and then the percentage of deuteration calculated (see Materials and Methods). The primary sequences of DnaB and DnaC, regions of secondary structure, the Walker A and B boxes, the arginine finger (arginine 442) of *E. coli* DnaB, and the initiator specific module (ISM) and AAA+ motifs of DnaC are also shown. The black bars and arrows represent α helices and β strands, respectively. In panel **C**, the N-terminal domain (NTD) of DnaB proposed to be involved in dimerization of DnaB (41–44), residues 135 and 141 required for interaction with primase (45,46), and its larger C-terminal domain (CTD) involved in nucleotide binding and hydrolysis and in interacting with DnaC (residues 295–304 and 431–435) correlate with the X-ray crystallographic structure of *Geobacillus kaustophilus* and *Geobacillus stearothermophilus* DnaB (8,10). The vertical bars represent the Walker A and B boxes and the arginine finger (arginine 442 of *E. coli* DnaB). Other functional domains shown are the helical hairpins (residue 119–148, 152–169), the linker helix (residue 182–198), and the domain that forms the hexamerization pocket (residue 264–308). For DnaC, its sites that interact with DnaB, the ISM, and the AAA+ motifs of its AAA+ domain are shown.

Differential HDX mass spectrometric analysis relies on the time-dependent increase in mass of a peptide fragment caused by deuterium incorporation via amide hydrogen exchange (40). The rate of exchange is influenced by local stability, accessibility to the solvent and structural changes. Hence, the amide hydrogens of a particular peptide often exchange at different rates. We performed HDX analysis to probe the conformational dynamics of DnaB complexed with DnaC in comparison with DnaB and DnaC alone. We prepared the individual proteins or the BC complex in a buffer containing ATP or ATPγS at 20°C and then diluted the samples 10-fold into the same buffer, but made with D_2_O. At the indicated times, samples were combined with chilled 1% formic acid to inhibit back exchange. Following online pepsin digestion and rapid separation on a reverse phase C18 analytical column, the peptides were analyzed by electrospray ionization-mass spectrometry (ESI-MS). As the peptides were compared under essentially identical conditions, the results were not corrected for back exchange, which was from 30–50% depending on the individual peptide. The overall sequence coverage of DnaB and DnaC peptides identified was 87% and 90%, respectively (Figure 1A, B).

### Conformational dynamics of DnaB helicase

Summarized above, several structural models describe that DnaB contains an NTD joined to its CTD via a linker helix (Figure 1C). The linker helix also associates with a site named the hexamerization pocket in the CTD of the adjacent DnaB protomer. The X-ray crystallographic structures of *G. stearothermophilus* DnaB bound to a truncated form of primase in which DnaB is a closed ring (8), or bound to ssDNA and GDP-AlF_4_ in which DnaB is an open spiral show an open triangular conformation of the NTDs (12). Two contiguous α helices of one protomer form a helical hairpin that interacts with the hairpin in the neighboring protomer to assemble a four-helix bundle in the dimerization of the respective NTDs (41–44). A recent study employed image reconstruction and refinement of DnaB and the BC complex visualized by cryo-EM (32). In the presence of ATP or ATP analogues (ATPγS, AMP-PNP), the helical hairpins form the four helix bundle of an N-terminal collar of DnaB that is either dilated or constricted.

The HDX results obtained for peptides of DnaB served as the control for the analysis of exchange rates for the BC complex, which are described later. We grouped the DnaB peptides by their rates of hydrogen/deuterium exchange. Consistent with their locations on the surface of a homology model of *E. coli* DnaB prepared using the X-ray crystallographic structure of *G. stearothermophilus* DnaB (PDB code 2R6A), peptides in the first group displayed fast exchange kinetics even at early time points (Supplementary Figures S1, S2A, S2B, S3). For other peptides, their slower deuterium incorporation that progressed up to 30 min may be explained by amide hydrogen bonding with other segments of DnaB that leads to intermediate exchange rates. The third group contains peptides that are at or near the dimer interface of DnaB, and exhibited either much slower or marginal exchange rates that indicate a more rigid local structure and strong hydrogen bonding. By comparison, peptides including one (267–281), which contains part of the hexamerization pocket (residues 264–308, (8)), remained largely un-deuterated, compatible with their buried locations in the model of *E. coli* DnaB. We note that only a few peptides had exchange rates at odds with their locations (see Supplementary Table S1). Together, these observations support the validity of the homology model.

### The N-terminal collar of DnaB is in an altered conformation in the BC complex

**Figure 2.**
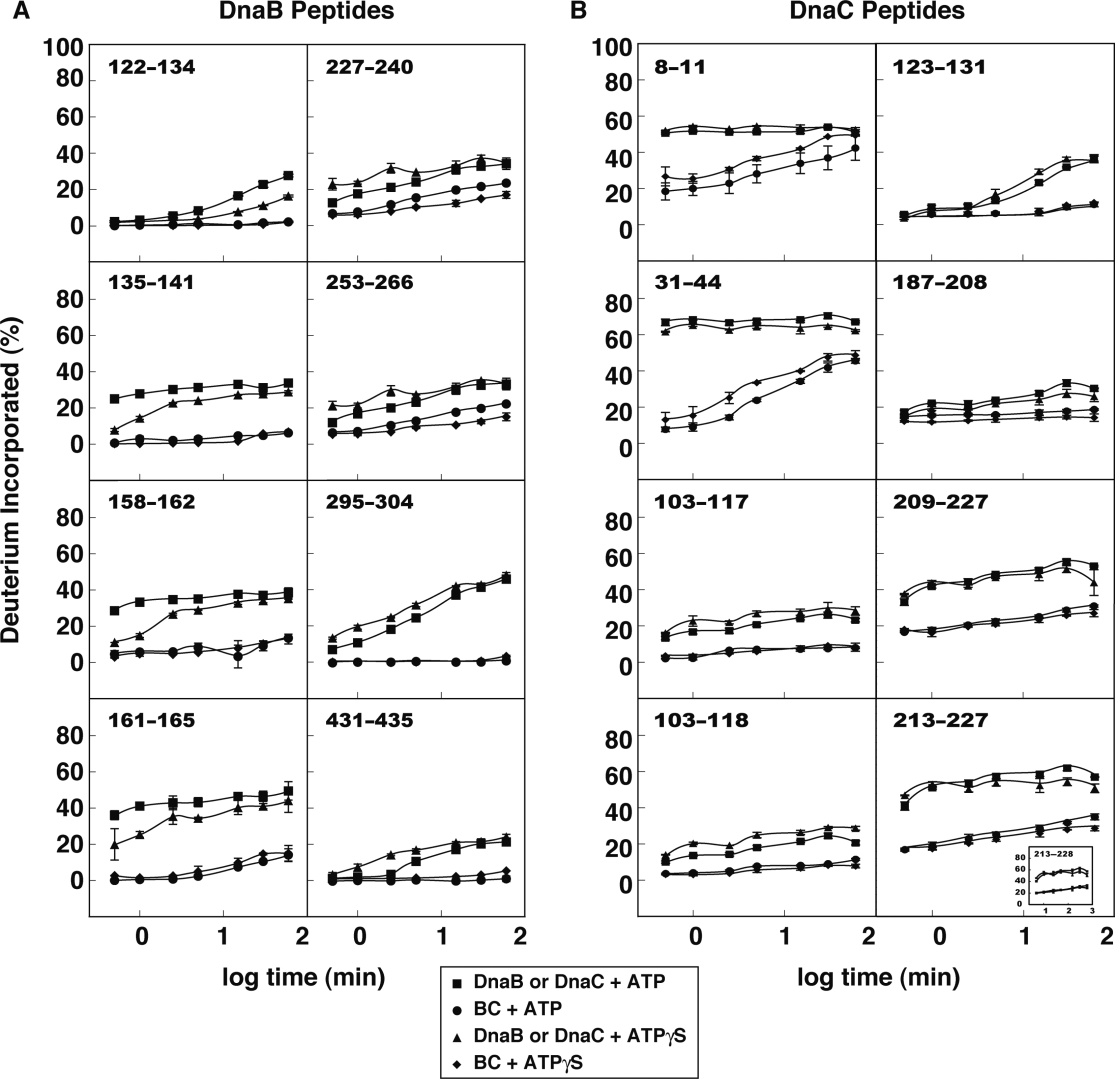
Deuterium incorporation into select peptides of DnaB and DnaC. The rates of deuteration of the respective peptides in the presence of ATP or ATPγS are shown. At least three independent sets of experiments with ATP were performed from which the standard deviation was calculated. With ATPγS, two independent experiments were performed from which the range has been plotted relative to each data point. The symbols for most time points obscure the error bars.

We then compared the HDX kinetics of peptides of DnaB when complexed with DnaC with that of DnaB alone (Figure 2A). This analysis identified both regions of DnaB that undergo conformational changes upon binding to DnaC, and a site that directly interacts in forming the BC complex. With ATP present, peptides (residues 122–134 and 135–141) located in one of the α-helices of the helical hairpin undergo exchange in the absence of DnaC, with a slower rate of exchange for the 122–134 peptide than for the 135–141 peptide that is part of the four-helix bundle (8,11). Substitution of ATP with ATPγS yields a slower rate of exchange at earlier time points, suggesting that ATP hydrolysis exposes the helical hairpin of each DnaB protomer to buffer. In contrast, deuterium exchange was severely inhibited for these peptides in the BC complex. Apparently, DnaC complexed to DnaB induces a conformational change that shields the helical hairpin of each DnaB protomer. Similarly, DnaB peptides containing residues 158–162 and 161–165 readily exchanged but not when DnaB was bound to DnaC. As mutational studies suggest that primase interacts with residues 135 and 141 of DnaB (45,46), these observations taken together with recent work showing that primase is able to interact with DnaB but not when it is bound by DnaC (Felczak and Kaguni, manuscript in preparation) strongly suggest that DnaC bound to DnaB occludes the helicase from primase.

### The role of the linker helix of DnaB in ring opening

**Figure 3.**
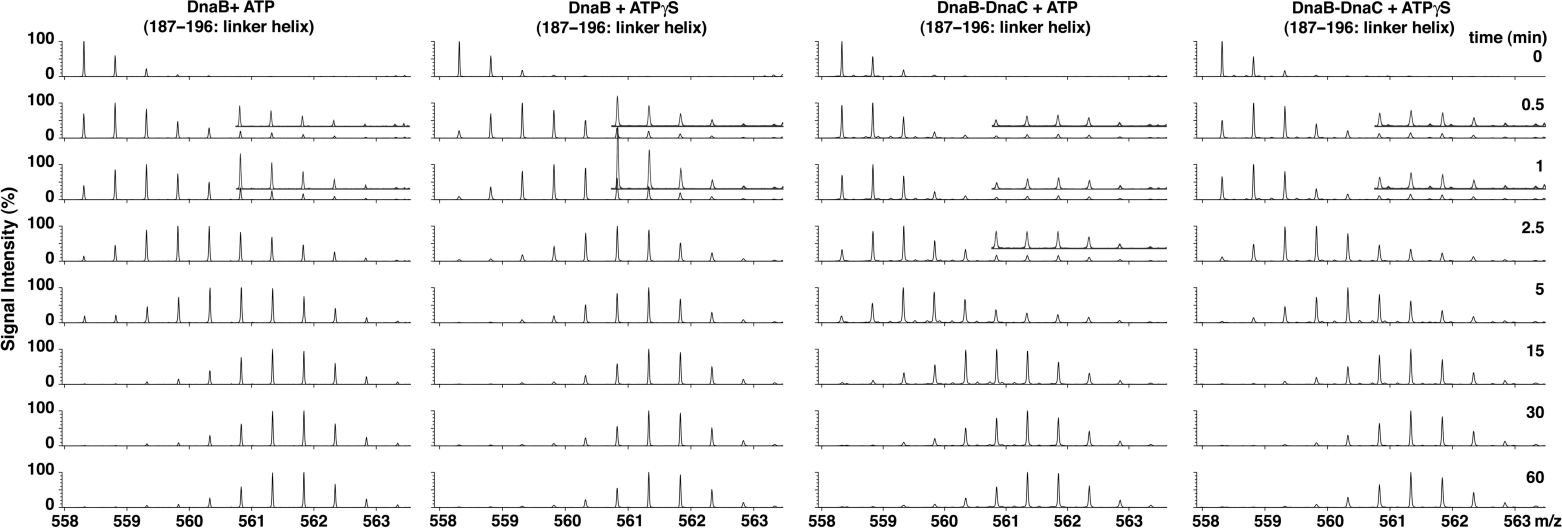
Bimodal exchange kinetics of the DnaB linker helix in the BC complex. Mass spectra show the abundances of ions with various mass/charge (*m*/*z*) values at the indicated times for the DnaB peptide (amino acids 187–196) in DnaB, or the BC complex in the presence of ATP or ATPγS. The bimodal isotopic distributions, which were also observed in raw mass spectra for lesser charge states (data not shown), indicate the presence of two populations of the DnaB linker helix. The vertical axes of peptides greater than *m*/*z* 561.346 (+2 charge state) of the respective spectra have been magnified three-fold.

Based on the homology model of *E. coli* DnaB, residues 182–198 of *E. coli* DnaB function as the linker helix, making intimate contact with residues in the hexamerization pocket (amino acids 264–308) in the CTD of the neighboring DnaB protomer (12). For single-stranded DNA to enter into the interior of DnaB, the single discontinuity in a DnaB hexamer must involve the dissociation of the linker helix from the neighboring DnaB protomer that is expected to lead to increased rates of hydrogen/deuterium (H/D) exchange at this unique interface. Remarkably, our experiments with the BC complex in buffer containing ATP or ATPγS indicate that a peptide (residues 187–196), which represents the behavior of the linker helix, displays a striking bimodal H/D exchange pattern at early time points (0.5–2.5 min, Figure 3). At 0.5 and 1 min, the majority of this peptide (85.5 ± 0.5% and 84.5 ± 0.7%, respectively) constitutes a low-mass envelope of the un-exchanged population whereas a small fraction (14.5 ± 0.1% and 17.5 ± 2.3%, respectively) occupies a high-mass envelope. To emphasize this bimodal distribution compared with the pattern seen with DnaB alone, the vertical axes of peptides greater than *m*/*z* 561 of the respective spectra have been magnified three-fold. Without correcting for merging of the low mass population into the high mass population with increasing time, the ratios of these populations suggests an open ring conformation in which one of every six DnaB protomers of each BC complex is separated from its neighbor. Studies of P4 helicase of bacteriophage ϕ8 revealed a similar bimodal exchange pattern that was attributed to ring opening (47). In contrast and as expected for a closed ring, only the low-mass envelope was observed for this peptide with DnaB alone at these time points.

DnaB has been described as a closed ring in the absence of DnaC (8–10). Hence with DnaB alone, we expected limited H/D exchange for this linker helix peptide because of its association with the CTD of the neighboring DnaB protomer. However, we did not find that it was shielded from exchange, but becomes deuterated to form the high mass population (Figure 3). Compared with ATP, its faster rate of deuteration in buffer containing ATPγS (0.5–5 min) suggests that the linker helix is more exposed, and that ATP hydrolysis is not required. This behavior suggests that the linker helix transiently dissociates from the neighboring DnaB protomer in the absence of DnaC. Considering the peptide's bimodal distribution in the BC complex, DnaC evidently traps DnaB as an open ring.

### DnaC alters the orientation of the Walker A box of DnaB

In the closed ring structures of DnaB, the Walker A box is surface-exposed (8–10). In agreement, a DnaB peptide (residues 227–240) that carries the Walker A box and another nearby peptide (residues 253–266) undergo exchange in the absence of DnaC (Figure 2A). In the BC complex, the rate of exchange of these peptides is substantially reduced. On the basis of fitting of atomic structures into a cryo-EM reconstruction of the BC complex, the Berger laboratory suggested that the binding of DnaC positions the AAA+ domain of DnaB in a conformation appropriate for nucleotide binding (32). Our results support this notion. The enigma is that DnaC when bound to DnaB inhibits its DNA-dependent ATPase activity (16,24). Perhaps the binding of DnaC leads to reorganization of DnaB's RecA domain so that it is poised to hydrolyze ATP, but its conformation is imperfect. We presume that an inducing signal adjusts the spatial arrangement of critical residues, leading to ATP hydrolysis.

### DnaC interacts with specific residues of DnaB to form the BC complex

An objective was to understand how DnaC interacts with DnaB to form the BC complex. In the absence of DnaC, the moderate exchange rate of DnaB peptides containing amino acids 295–304 and 431–435 contrasts with their greatly reduced rates when DnaC was bound (Figure 2A). These results suggest that these regions interact with DnaC. To test this idea, we constructed plasmids encoding alanine substitutions in these portions of DnaB, and measured their ability to complement a temperature-sensitive *dnaB* mutant (Supplementary Figure S4A, B). Compared with others, the alleles encoding I297A, L304A and E435A substitutions were defective in complementing the *dnaB* mutant at non-permissive temperature under conditions in which the *dnaB*^+^ plasmid was active. We also used an ELISA assay that measures the direct interaction of DnaB with DnaC, and showed that the E435A substitution blocks this interaction and also inactivates DnaB function in DNA replication of an *oriC*-containing plasmid whereas other substitutions (D291A, R296A and K431A) do not (Supplementary Figure S4C, D). Supported by observations described below, our results identify critical DnaB residues that DnaC recognizes to form the BC complex.

### Two α-helices near the N-terminus of DnaC interact with DnaB

By velocity sedimentation analysis in buffer containing magnesium ion and either ATP or ADP, DnaC is a monomer (48,49). Its affinities for ADP, ATP and ATPγS are similar (26,27). With ATP or ATPγS, our HDX analysis revealed that two peptides of DnaC (amino acids 8–11 and 31–44), which reside in the first two α helices predicted by secondary structure analysis (PSIPRED (50), data not shown), exhibited fast exchange kinetics (Figure 2B, Supplementary Table S1B). Many peptides in its AAA+ domain behaved similarly, indicating their exposed state, whereas others displayed a range from moderate to negligible exchange rates (Supplementary Table S1B).

**Figure 4.**
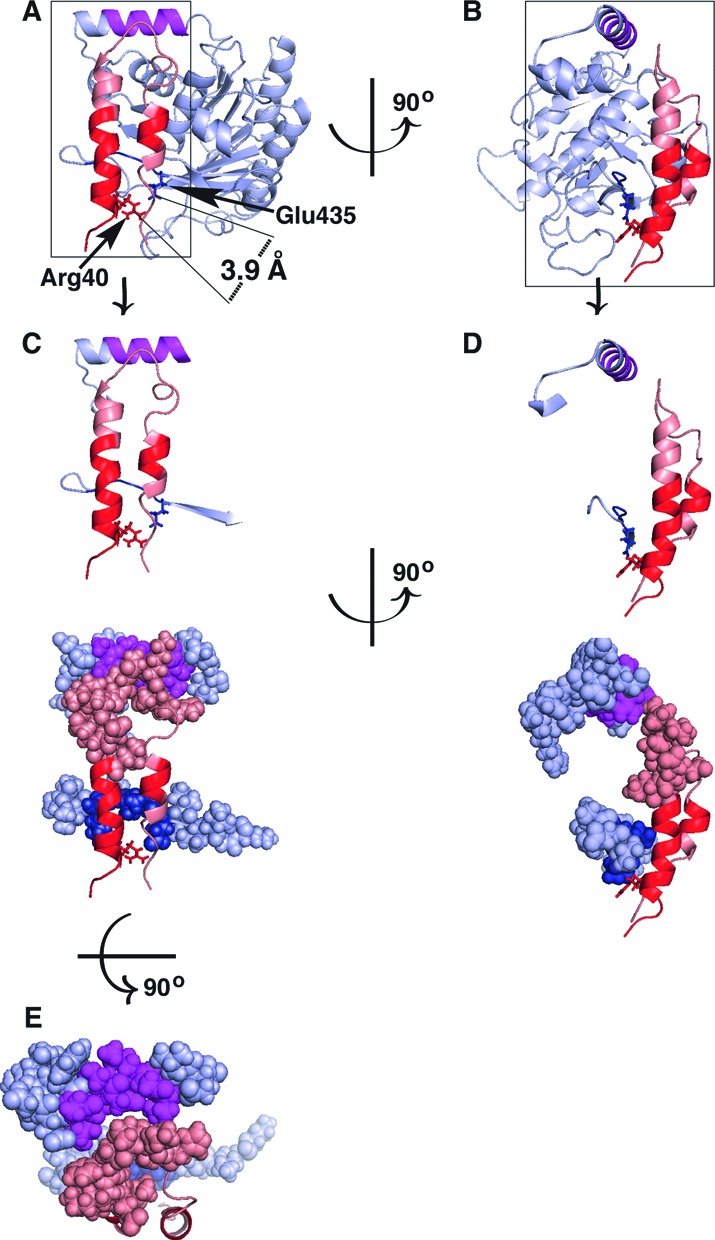
Amino acids 8–11 and 31–44 of DnaC interact with residues 431–435 of DnaB. Homology models of *E. coli* DnaB and DnaC built using MODELLER (51,52) with predicted α-helices in the N-terminal region (residues 1–64; (33), PSIPRED) were docked by simultaneously placing DnaC residues 8–11 and 31–44 in close proximity to DnaB residues 431–435 and fitting the resulting model into the cryo-EM density of the BC complex. In Panel **A** and **B**, the C-terminal portion of DnaB (residues 201–462) is shown in light blue with residues 431–435 in darker blue and 295–304 in magenta. Residues 1–44 of DnaC are shown in salmon, with segments containing amino acids 8–11 and 31–44 in red. Panel C–E are different views of the interacting regions of DnaB (residues 284–304 and 426–440) and DnaC (residues 1–44). The figures were prepared using PyMOL (The PyMol Molecular Graphics System, version 1.7.4). Please also see PyMol session files in Supplementary Information.

With either adenine nucleotide, DnaC peptides bearing amino acids 8–11 and 31–44 displayed greatly reduced exchange kinetics when complexed with DnaB (Figure 2B). These results corroborate earlier mutational studies, which showed that substitutions in these segments block the binding of DnaC to DnaB, and also severely inhibit its DNA replication activity (33). To provide an understanding of how these sites interact with DnaB, we docked DnaC onto DnaB using molecular modeling (51,52). Homology models of *E. coli* DnaB and DnaC were prepared using the X-ray structures of *G. stearothermophilus* DnaB (PDB code 2R6A) and the AAA+ domain of *G. kaustophilus* DnaI (PDB code 2W58) as templates (8,53). DnaB was built initially as a hexamer based on the 2R6A structure, and DnaC as three dimers based on two monomers in the asymmetric unit of the crystal, and on the parallel dimer interface of the AAA+ domain seen in *A. aeolicus* DnaA (PDB code 2HCB), a DnaC paralog (54). Because *E. coli* DnaC has two predicted α-helices in its N-terminal region (residues 1–64; (33), PSIPRED) attached to its AAA+ domain that are absent in the 2W58 template structure, we modeled the N-terminal region initially in an extended conformation to avoid clashing in the DnaC dimer. Guided by the experimental data, the DnaC dimers were then docked with DnaB by forming contacts between residues 8–11 and 31–44 of DnaC and residues 431–435 of DnaB (Figure 4). The CTD of DnaB and the AAA+ domain of DnaC were held rigid while allowing the helices near the N-terminus of DnaC to adjust their positions. The model was then further refined by docking DnaB and DnaC into the cryo-EM density map of the BC complex (32). The NTD of DnaB (residues 1–170) and residues 175–195 that bear the linker helix (residues 182–198) were allowed to move relative to the CTD of DnaB. Each protomer of DnaB and DnaC was also allowed to move independently to optimize the fit into the EM density. An outcome of this model is that the loop connecting two α-helices that contain residues 8–11 and 31–44 shields residues 295–304 of DnaB (Figure 4). Thus, this structure explains the negligible exchange of residues 295–304 in the BC complex, and the defective complementation of the *dnaB*(Ts) mutant by the I297A and L304A substitutions. Taken together, these observations validate the model.

### Conformational changes of DnaC's AAA^+^ motifs upon binding to DnaB

The AAA+ motifs of DnaC act in ATP binding, which is necessary for DnaC function. As evidence, a mutant DnaC of *E. coli* carrying a K112R substitution of the conserved Walker A lysine (Lys112 in GKPGTGKN from residues 106–113) is deficient in both ATP binding and helicase loading at *oriC* (27). Separate work characterized mutant DnaCs of *E. coli* that failed to interact with DnaB (33). Some carry amino acid substitutions in and near the Walker A box (S101I, P108S, H114G and H114R), demonstrating that ATP binding is needed for DnaC activity in the BC complex. Together, these findings underscore the essential role of ATP binding by DnaC for its function.

In light of the above results, we analyzed the kinetics of H/D exchange of peptides in the AAA+ domain of DnaC. In presence of ATP or ATPγS, H/D exchange by those bearing the Walker A motif (peptides 101–115, 103–117 and 103–118) and an adjacent segment (peptides 118–123, 123–131 and 124–131) was inhibited when DnaC was complexed to DnaB (Figure 2B, Supplementary Figure S5). These results support the idea that the Walker A motif and other parts of DnaC (see below) reorganize upon interacting with the adenine nucleotide to mask their ability to exchange. Hence, ATP has a marked effect despite studies showing that formation of the BC complex does not require a nucleotide (26,27).

The box VII and sensor I motifs of AAA+ proteins contain a conserved arginine and asparagine, respectively (55,56). For those AAA+ proteins whose X-ray structures are known, the residue in box VII named the arginine finger interacts with the γ-phosphate of ATP in the binding pocket formed between adjacent protomers of an oligomer, and is proposed to coordinate a conformational change with ATP hydrolysis. The box VII motif of *E. coli* DnaC has two conserved arginines (Arg216 and Arg220) that are essential (6,28). Arg220 is thought to be the arginine finger. The conserved asparagine of DnaC's sensor I motif (residues 201–203; LTN) presumably serves to detect nucleotide binding or hydrolysis by hydrogen bonding with the terminal phosphate of ATP (57,58). Like the peptides containing the Walker A box, peptides bearing the box VII motif (209–227, 213–227 and 213–228; Figure 2B) and sensor I (187–208; Figure 2B) were protected from deuterium exchange when DnaC was complexed to DnaB. The behavior of these peptides correlates nucleotide binding by DnaC with its interaction with DnaB.

### The initiator specific motif (ISM) of DnaC shows a bimodal exchange pattern when DnaC is bound to DnaB

A subset of AAA^+^ proteins that include DnaC and DnaA contain two extra α-helices between the Walker A and B boxes that form a domain named the ISM (28,59–60). In the proposed right-handed helical structure of the BC complex (32), the first α helix of the ISM in the presence of ATP is suggested to pack against the neighboring DnaC molecule, causing the spiral assembly of DnaC protomers. As M139E and F146D substitutions of hydrophobic residues in the first ISM α helix of *E. coli* DnaC greatly reduced the ability of plasmids encoding the alleles to complement a *dnaC2* mutant at non-permissive temperature (28), these observations support the model in which each ISM contacts an adjacent protomer of a DnaC oligomer.

**Figure 5.**
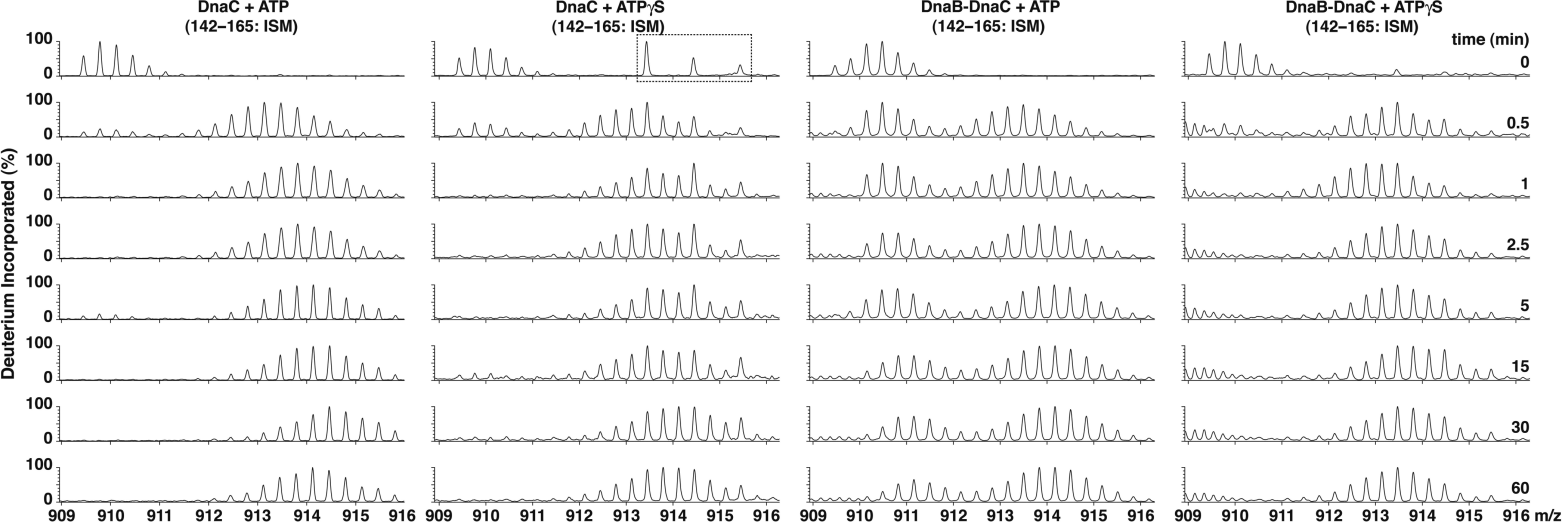
Bimodal exchange kinetics of the initiator specific module of DnaC in the BC complex. Mass spectra distributions at the indicated times for the DnaC peptide (amino acids 142–165, *m*/*z* 909.465, +3 charge state) in DnaC or the BC complex in the presence of ATP or ATPγS are shown. The bimodal isotopic distributions indicate the presence of two populations of the initiator specific module of DnaC. In the set of spectra of DnaC with ATPγS, the peptides in the dashed box that are absent in the other series of spectra originate from a contaminant in the preparation of DnaC used for this experiment (data not shown).

Our HDX analysis identified a peptide (amino acids 142–165, Figure 5) that contains most of the ISM of DnaC (residues 136–162). Without DnaB, its similar rate of deuterium uptake with either ATP or ATPγS correlates with the monomeric state of DnaC (48,49). Remarkably, this peptide showed a striking bimodal isotopic distribution when DnaC was bound to DnaB in the presence of ATP but not ATPγS (center-right and right panels, Figure 5). As peptides 154–162 and 160–166 did not show a bimodal pattern (Supplementary Figure S5), we attribute the difference to residues 142–153, which carries half of the first ISM α-helix and the loop that follows. The bimodal distribution pattern reflects a protected population, and another that exchanges more rapidly like that seen with DnaC alone with ATP or ATPγS. Despite evidence showing that BC complex formation does not require ATP (26,27), the effect of ATP but not ATPγS for protection indicates different conformations of DnaC in the BC complex. One formed with ATPγS does not have each ISM protected whereas the protected population with ATP presumably arises via contact of the ISM of one DnaC molecule with its neighbor. Considering the comparable abundance of both populations, this neighboring DnaC protomer apparently only interacts with its partner. These results agree with cryo-EM analysis of the BC complex in the presence of ATP wherein DnaC appears as sets of dimers bound to DnaB (21). As DnaC appears to trap DnaB as an open ring, one of the gaps separating each DnaC dimer enables passage of the single-stranded DNA into the interior of the BC complex where the DNA will become bound. Although DnaC is a weak ATPase (27), the poor protection of the ISM in the presence of ATPγS suggests that nucleotide binding alone is insufficient, and implicates a role of ATP hydrolysis. As the proportion of the constricted or dilated N-terminal collar of *E. coli* DnaB is about equal with ATP or ATPγS (11), the exchange behavior of the ISM does not appear to correlate with these DnaB forms.

## DISCUSSION

### DnaC controls the access of DnaB to primase

At the stage of replication initiation, DnaA loads one BC complex on each separated strand of the unwound region within *oriC* (61). Earlier, we reported that conditions suitable for primer formation by primase cause DnaC to dissociate from DnaB (6). An attractive model is that the binding of primase to the NTD of DnaB at *oriC* induces a conformational change in the CTD of DnaB that leads to the release of DnaC and activation of the helicase. Of interest, our HDX results indicate that DnaC bound to DnaB causes peptides of DnaB's NTD that are recognized by primase to become inaccessible to exchange. These observations, which were obtained in the absence of *oriC*, strongly suggest that the surface of DnaB that interacts with primase is occluded. Hence, prior to binding of the BC complex at *oriC*, DnaC renders DnaB unable to interact with primase. After the BC complex has loaded at *oriC*, the interacting region on DnaB apparently becomes available.

Recent cryo-EM analysis revealed alternate conformations of the NTD of *E. coli* DnaB that were described as dilated or constricted (11). Biochemical analysis of mutants constructed to form the dilated or constricted conformation showed that, in the absence of DnaC, the former was able to interact with primase as measured indirectly by RNA primer synthesis whereas the latter was essentially inert. These observations together with our finding that DnaC bound to DnaB occludes the site in its NTD recognized by primase suggest a biochemical mechanism to describe how DnaC controls the access of DnaB to primase.

### ATP and the BC complex

Despite earlier studies showing that ATP is unnecessary for BC complex formation (26,27), we discovered that ATP profoundly affects the conformation of DnaB and DnaC in the BC complex. Compared with DnaC alone, we found that its Walker A box, box VII and sensor I motif are protected from exchange in the BC complex. Moreover, DnaC in the BC complex causes reorganization of DnaB's Walker A box so that it is not susceptible to hydrogen/deuterium exchange, consistent with the notion that the Walker A box is now almost suitably aligned to support ATP hydrolysis. If so, DnaB is poised to act as a DNA helicase after DnaC dissociates.

### DnaC traps DnaB as an open ring

The behavior of the linker helix of DnaB indicates that the interface separating DnaB protomers in the DnaB ring transiently opens, and that DnaC arrests DnaB as an open ring. HDX analysis of the BC complex combined with previous studies of mutant DnaCs show that two sites (residues 8–11 and 31–44) in DnaC interact with a site (residues 431–435) in DnaB. That Glu435 of DnaB interacts with DnaC supports this conclusion (Supplementary Figure S4, (33)). Of interest, Glu435 and Arg40 of DnaC are separated by about 3.9 Å in the BC complex (Figure 4A), so these residues may form a salt bridge. To further our understanding of how the BC complex forms, we used molecular modeling to integrate the structural information of DnaB and DnaC into the EM reconstruction of the BC complex with our identification of the sites in DnaB and DnaC that interact. The sites predicted to reside in two α helices of DnaC by secondary structure analysis (PSIPRED (50), data not shown) fit well against DnaB in the open ring DnaB model to support the idea that DnaC exploits the open ring form of DnaB (Figure 4). Its binding blocks ring closure. This mechanism contrasts with the suggestion that the binding of DnaC to DnaB causes the DnaB ring to open (32). Like DnaB, the sliding clamps (β and gp45) of DNA polymerase III and bacteriophage T4, respectively, are ring molecules. As the rings are open in solution (62,63), a proposed pathway of clamp loading involves capturing the open clamp by the DNA-bound clamp loader and then loading it onto DNA (64,65). By analogy, DnaA loads the BC complex at *oriC* in which DnaB exists in the open ring state.

### The hexamerization pocket of DnaB

X-ray structures show that the hexamerization pocket, a hydrophobic surface formed by three α-helices, cradles the linker helix of the adjacent protomer in the DnaB hexamer (residues 264–308 and 182–198, respectively, for *E. coli* DnaB). The linker helix peptide (187–196) displays bimodal exchange kinetics, reflecting opening of the DnaB ring. We expected but failed to detect a similar exchange pattern for peptides 267–281, and 277–289 that compose the hexamerization pocket. Perhaps ring opening causes only a few amide hydrogens in these peptides to exchange, which is difficult to detect (66,67). A part of this pocket, residues 295–304 did not show bimodal kinetics, but their occlusion by DnaC provides an explanation. We also note that the segment from amino acids 290–294 was not detected in our analysis. It is possible that these residues would have indicated ring opening.

## Supplementary Material

SUPPLEMENTARY DATA
