# Supplementary material for: DnaC traps DnaB as an open ring and remodels the domain that binds primase
Source: Nucleic Acids Res. 2015 Sep 29;44(1):210–20. doi: 10.1093/nar/gkv961 (PMC4705694; doi:10.1093/nar/gkv961)
Supplement: SUPPLEMENTARY DATA [file supp_44_1_210__index.html]

DnaC traps DnaB as an open ring and remodels the domain that binds primase — DnaC traps DnaB as an open ring and remodels the domain that binds primase — SUPPLEMENTARY DATA 

# DnaC traps DnaB as an open ring and remodels the domain that binds primase

## SUPPLEMENTARY DATA

- SUPPLEMENTARY DATA
- SUPPLEMENTARY DATA
- SUPPLEMENTARY DATA
- SUPPLEMENTARY DATA
- SUPPLEMENTARY DATA
- SUPPLEMENTARY DATA
- SUPPLEMENTARY DATA
- SUPPLEMENTARY DATA
- SUPPLEMENTARY DATA
