# Supplementary material for: DnaC traps DnaB as an open ring and remodels the domain that binds primase
Source: Nucleic Acids Res. 2015 Sep 29;44(1):210–20. doi: 10.1093/nar/gkv961 (PMC4705694; doi:10.1093/nar/gkv961)
Supplement: SUPPLEMENTARY DATA [file supp_gkv961_nar-01994-m-2015-File007.pdf]

DnaC traps DnaB as an open ring and remodels the domain t hat binds primase

Sundari Chodavarapu, A. Daniel Jones, Michael Feig and Jon M. Kaguni

Supplementary Information

Figure S1. Homology models of *E. coli* DnaB and DnaC were constructed using MODELLER (1,2) as described in the legend of Figure 4. The models were then fitted into the cryo-EM density of the BC complex (3). Most of DnaB is shown in light blue with residues 122-134 and 135-141 that correspond to the helical hairpins in orange, 187-196 (linker) in green, 295-304 in magenta, and 431-435 in dark blue. DnaC is salmon-colored, but amino acids 8-11 and 31-44 are in red. As indicated, views of DnaB or the BC complex show the NTD of DnaB, side view and the CTD of DnaB or DnaC. The figures were prepared using PyMOL (The PyMol Molecular Graphics System, version 1.7.4).

Figure S2. Using homology models of *E. coli* DnaB or DnaC alone and in the BC complex, the relative surface exposure of a sliding 5 amino acid segment of DnaB or DnaC was compared with hydrogen/deuterium exchange data. Surface exposure was calculated from the solvent-accessible surface using the COOR SURF command in CHARMM with a 2 Å probe radius. Error values indicate the standard deviation with respect to averaging SASA values over hexameric subunits. The hydrogen/deuterium exchange data for the 5 min time points were scaled to match the range of SASA values. The relative surface exposure of five residue segments of DnaB is shown compared with H/D exchange data for peptides obtained with DnaB by itself and in the BC complex in (A). The comparison in (B) is for DnaC alone and in the BC complex.

Figure S3. Deuterium uptake of peptides of DnaB alone or in the BC complex in the presence of ATP or ATPγS was measured at the times indicated as described in the Materials and Methods. For those shown, DnaC did not appreciably affect the exchange kinetics.

Figure S4. Mutant DnaBs bearing I297A, L304A, and E435A substitutions are defective in complementation of a *dnaB*(Ts) mutant in vivo; E435A is defective in DNA replication in vitro, and in interacting with DnaC. (A) *E. coli* RM84 (genotype: *dnaB22*(Ts) F^- Str^r (λ112)) was transformed by electroporation with plasmid pET3c or derivatives that encode the respective *dnaB* alleles. After plating serial dilutions on LB medium supplemented with ampicillin (100 μg/ml), colony formation was measured following overnight incubation at 30°C and 42°C. The efficiency of transformation at 30°C ranged from 0.3 – 3 x 10^9 per μg of plasmid DNA. Plasmids encoding wild type DnaB, and other substitutions were active in vivo as indicated by complementation of the temperature-sensitive phenotype of RM84 (*dnaB22*) at 42°C. Although the plasmid-borne *dnaB* alleles are downstream from a promoter recognized by bacteriophage T7 RNA polymerase, which is absent in the host strain, the ability of the *dnaB*$^+$ gene to complement the *dnaB*(Ts) host suggests that expression of the *dnaB* alleles is dependent on recognition of an upstream promoter by *E. coli* RNA polymerase. In contrast, plasmids expressing I297A, L304A, and E435A substitutions were defective as indicated by the lower plating efficiency (A) or much smaller colonies at 42°C (B), indicating that these mutant proteins are inactive in DNA replication in vivo. (C) Assays of in vitro DNA replication of an *oriC*-containing plasmid (M13*oriC*2LB5, 200 ng) were performed with highly purified proteins (4), including DnaB or the respective mutants as indicated. Wild type DnaB, D291A, R296A, and K431A) were purified from *E. coli* BL21(DE3) pLysS *fhuA2 [lon] ompT gal (λDE3) [dcm]* Δ*hsdS* induced to express

the respective *dnaB* alleles carried in pET3c essentially as described (4). E435A was purified from *E. coli* Lemo21 *fhuA2 [lon] ompT gal (λDE3) [dcm] ΔhsdS/ pLemo*(Cam^R). Other mutant DnaBs were not examined either because they were active in complementation of RM84, or were insoluble when overproduced. Incubation to measure DNA synthesis was at 30°C for 20 min, and acid-insoluble radioactivity was measured by liquid scintillation spectroscopy. (D) Enzyme-linked immunosorbent assays were performed by immobilizing the indicated amounts of DnaC or BSA in Buffer B (25mM Hepes-KOH pH 7.6, 10% (v/v) glycerol, 20 mM NaCl, 5 mM $MgCl_2$, 2mM DTT and 1 mM ATP) followed by incubation for 1 hr at room temperature. After washing the wells with the above buffer supplemented with 0.005% Tween 20 and 4% nonfat milk, the wells were incubated with this buffer for 1 hr at room temperature. DnaB or the indicated mutants purified as described above (200 ng in 100 μl of Buffer B) were then added followed by incubation for 1 hr at room temperature. After washing the wells with Buffer B supplemented with 0.005% Tween 20 and 2% nonfat milk, affinity-purified antibody that similarly recognizes wild type DnaB and the mutants (data not shown) was added and the plate was incubated overnight at 4°C. After successive washing to remove unbound antibody, immune complexes were detected colorimetrically at 490 nm with goat anti-rabbit antibody conjugated to horseradish peroxidase.

Figure S5. Deuterium incorporation of DnaC peptides in the presence of ATP or ATPγS. Reactions contained either DnaC only or the BC complex. For peptides containing amino acids 44-50, 85-94, 91-94 (a subfragment of the former peptide), and 118-123, an adenine nucleotide or DnaB dramatically affected their exchange kinetics. With ATP, the exchange rate was marginal compared with a much faster rate with ATPγS. Inasmuch as DnaC is a weak ATPase

(5), these results suggest that ATP hydrolysis causes a conformational change. When DnaC was complexed to DnaB in the presence of either nucleotide, these peptides displayed a nominal rate of exchange at early time points, indicating that the binding of DnaB alters DnaC's structure to lead to their protection. The panel displaying the DnaC peptide bearing residues 12-29 shows its exchange kinetics and that of an unknown peptide that comprises 40-50 % of the total.

Supplementary Tables

Table S1A: Exchange Kinetics of DnaB Peptides

	Fast Exchange	Moderate Exchange	Marginal Exchange	No Exchange
N-terminal domain	3-33 [b]<br>109-121<br>110-121 [a]<br>135-141<br>142-155<br>158-162 [c]<br>161-165 [c]	92-97<br>122-134<br>123-134 [a, b]<br>197-214	33-38<br>44-53<br>72-84<br>76-84	33-43<br>54-60<br>58-69 [b]<br>85-91 [c]
Linker helix		187-196 (bimodal pattern)		
C-terminal domain	347-360<br>348-355<br>348-360 [a]<br>382-402 [b]<br>458-471<br>461-471	227-240<br>227-244 [a, b]<br>229-240 [a]<br>253-266<br>260-267 [b]<br>277-289 [b]<br>295-304<br>294-304 [a]<br>391-402 [b]<br>418-430 [b]<br>413-426 [a, b]<br>431-435<br>452-460 [b]	215-226<br>258-267<br>305-322<br>306-339 [b]<br>436-451	245-251 [c]<br>267-281 [b]<br>340-344 [b]<br>365-382 [b, c]

Table S1B: Exchange Kinetics of DnaC Peptides				
	Fast Exchange	Moderate Exchange	Marginal Exchange	No Exchange
N-terminal region	1-7 [b]<br>8-11<br>8-29 [b]<br>12-29 [b]<br>31-44<br>75-84 [b, d]	95-102<br>96-102 [a]	44-50 [d]<br>85-94 [d]<br>91-94 [d]	
AAA+ domain	142-165 (bimodal pattern)<br>154-165<br>167-187 [b]<br>171-186<br>209-227<br>213-227 [b]<br>213-228 [b]<br>229-236<br>237-245<br>229-245 [b]	118-123<br>123-131 [d]<br>124-131 [d]<br>132-141	101-115<br>103-117 [b]<br>103-118<br>160-166<br>187-208	166-170 [b]

[a] This DnaB peptide displayed essentially identical exchange kinetics as the peptide directly above, so its exchange kinetics have been omitted in Figure S1 and Figure S2.

[b] Compared with other peptides whose identities were established by amino acid sequence analysis by fragmentation and tandem mass spectrometry, this peptide was identified by comparing its experimental mass with its predicted mass.

[c] HDX data indicates that DnaB peptides carrying residues 85-91, 245-251 and 365-382 show very limited exchange rates, suggesting that they are buried. However, these segments are surface-exposed in the homology models of DnaB and the BC complex (Figure S1). In contrast, peptides containing residues 158-162 and 161-165 display fast exchange kinetics indicating that

this region is surface exposed, and show substantially reduced exchange kinetics only when DnaC is bound to DnaB (Figure 2A). In the homology models, this region is buried. Considering that the H/D exchange experiments reflect the dynamic behavior of DnaB, DnaC, and the BC complex, we attribute these discrepancies to the homology model of BC complex that was derived from the X-ray crystallographic structures of the respective homologues, which may not strictly conform with their native structures.

[d] In the presence of ATP, DnaC peptides bearing residues 123-131 and 124-131 displayed moderate exchange rates, which were reduced in the BC complex (Figure S5). Whereas these results suggest that these residues are only partially exposed in the absence of DnaB and become buried in the BC complex, they are surface-exposed in the homology models of DnaC and the BC complex. Likewise, DnaC peptides 44-50, 85-94, and 91-94 are surface-exposed in these models, but the limited exchange suggests that they are buried.

Supplementary References

1. Sali, A. and Blundell, T.L. (1993) Comparative protein modelling by satisfaction of spatial restraints. *J Mol Biol*, **234**, 779-815.
2. Fiser, A., Do, R.K. and Sali, A. (2000) Modeling of loops in protein structures. *Protein Sci*, **9**, 1753-1773.
3. Arias-Palomo, E., O'Shea, V.L., Hood, I.V. and Berger, J.M. (2013) The bacterial DnaC helicase loader is a DnaB ring breaker. *Cell*, **153**, 438-448.
4. Makowska-Grzyska, M. and Kaguni, J.M. (2010) Primase directs the release of DnaC from DnaB. *Mol Cell*, **37**, 90-101.
5. Davey, M.J., Fang, L., McInerney, P., Georgescu, R.E. and O'Donnell, M. (2002) The DnaC helicase loader is a dual ATP/ADP switch protein. *EMBO J*, **21**, 3148-3159.

Figure S1

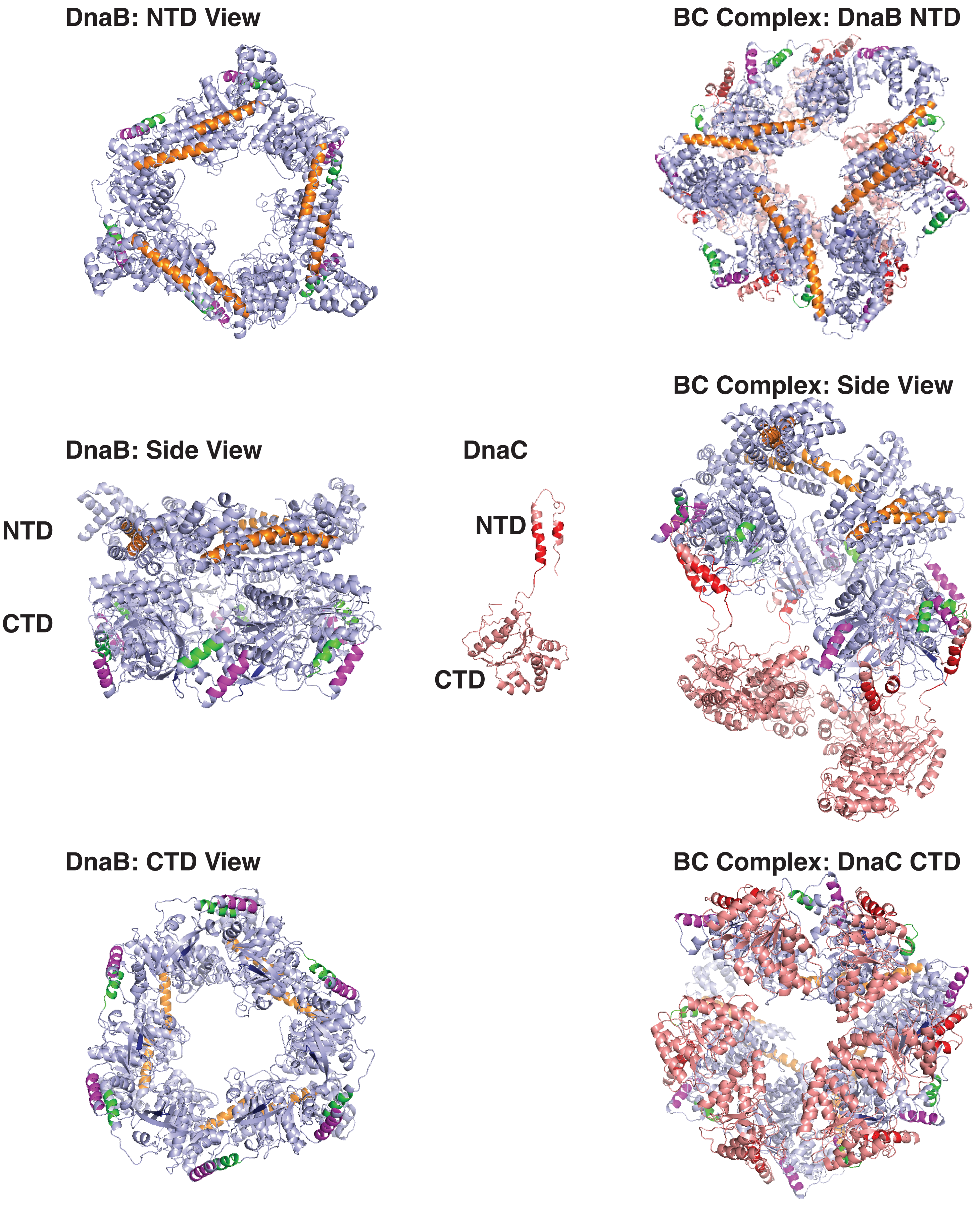
DnaB: NTD View
BC Complex: DnaB NTD
BC Complex: Side View
DnaB: Side View
DnaC
NTD
CTD
NTD
CTD
DnaB: CTD View
BC Complex: DnaC CTD

Figure S2A

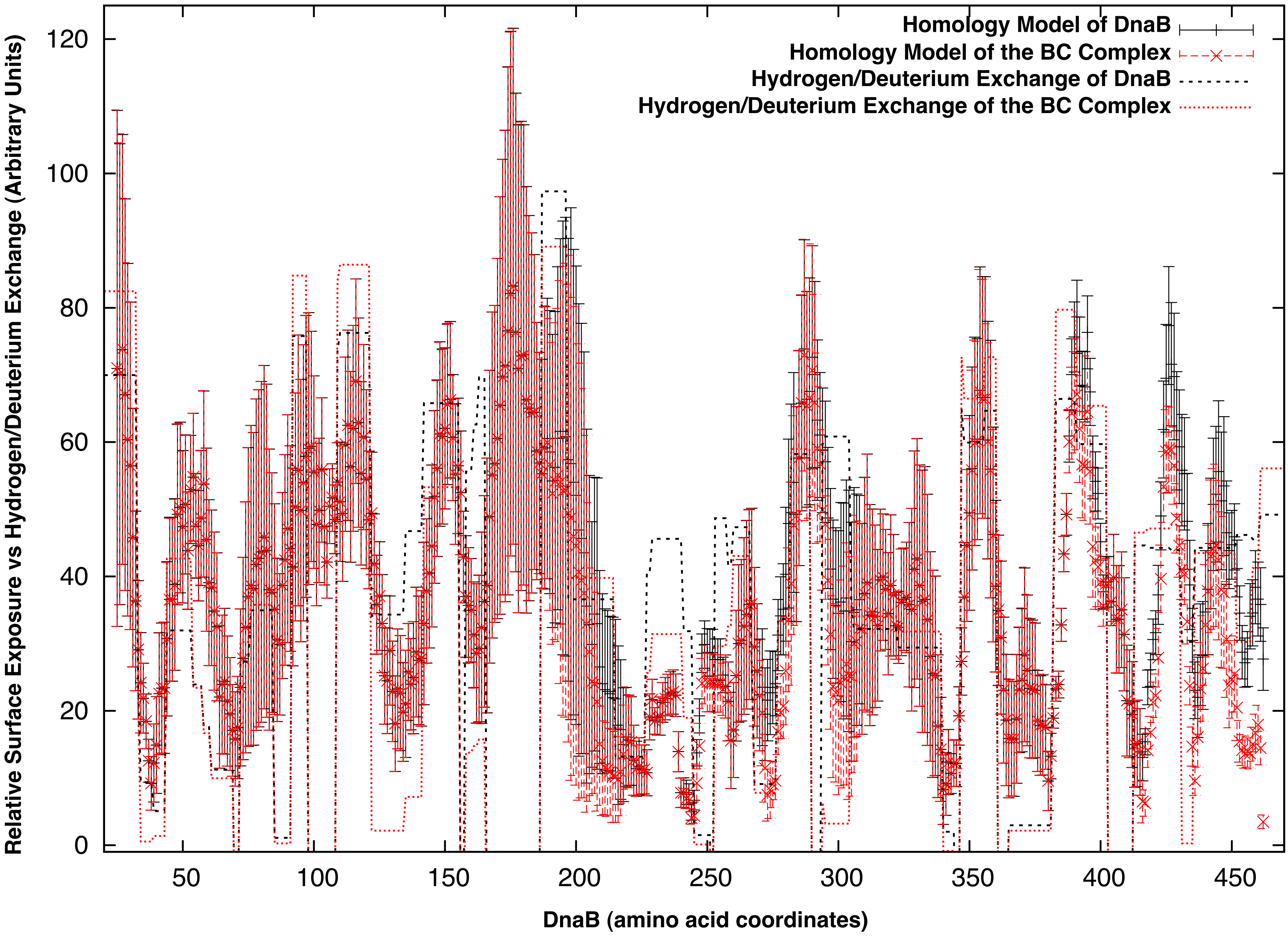
Homology Model of DnaB
Homology Model of the BC Complex
Hydrogen/Deuterium Exchange of DnaB
Hydrogen/Deuterium Exchange of the BC Complex
Relative Surface Exposure vs Hydrogen/Deuterium Exchange (Arbitrary Units)
DnaB (amino acid coordinates)
0
20
40
60
80
100
120
50
100
150
200
250
300
350
400
450

Figure S2B

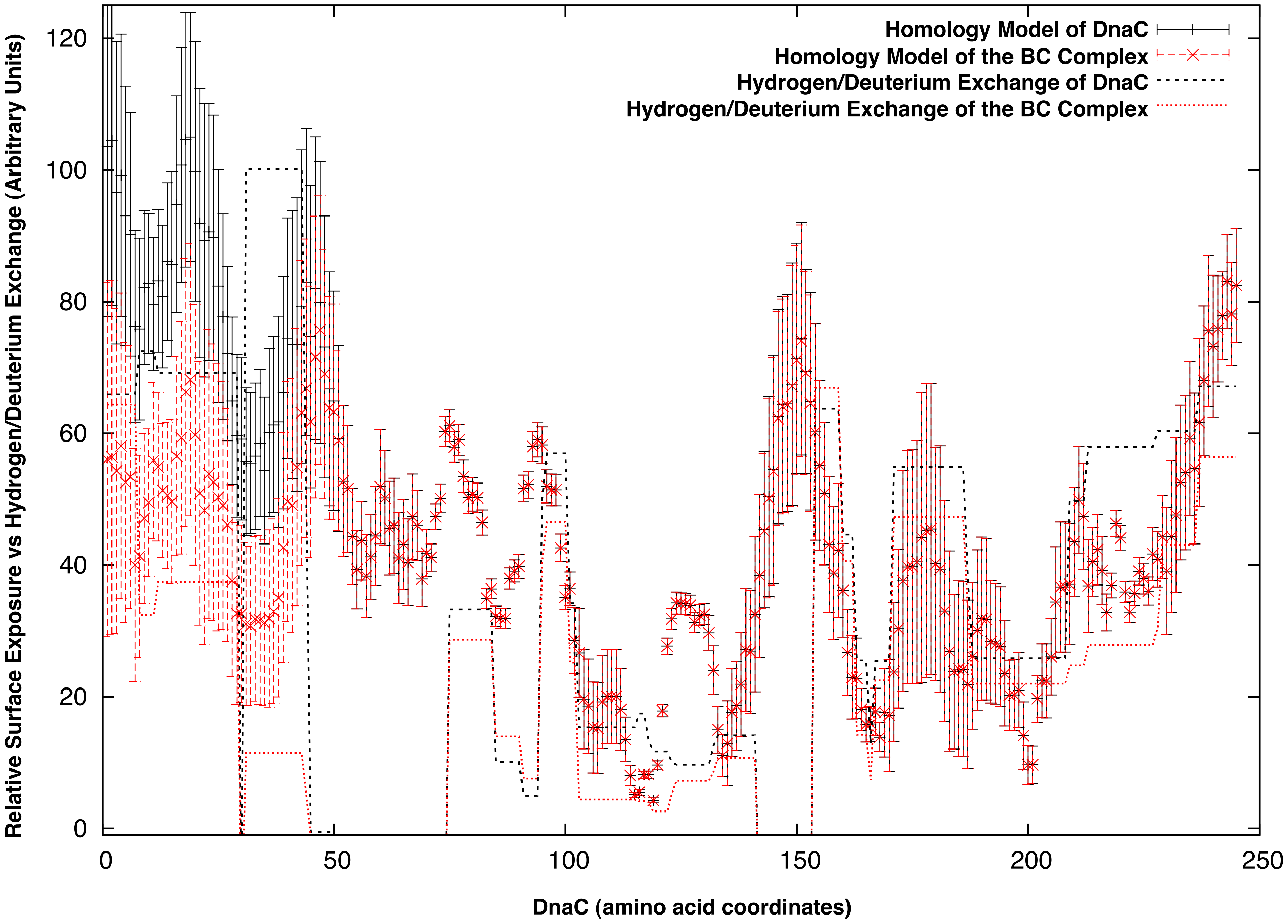

Homology Model of DnaC
Homology Model of the BC Complex
Hydrogen/Deuterium Exchange of DnaC
Hydrogen/Deuterium Exchange of the BC Complex
Relative Surface Exposure vs Hydrogen/Deuterium Exchange (Arbitrary Units)
DnaC (amino acid coordinates)
0
20
40
60
80
100
120
0
50
100
150
200
250

Figure S3

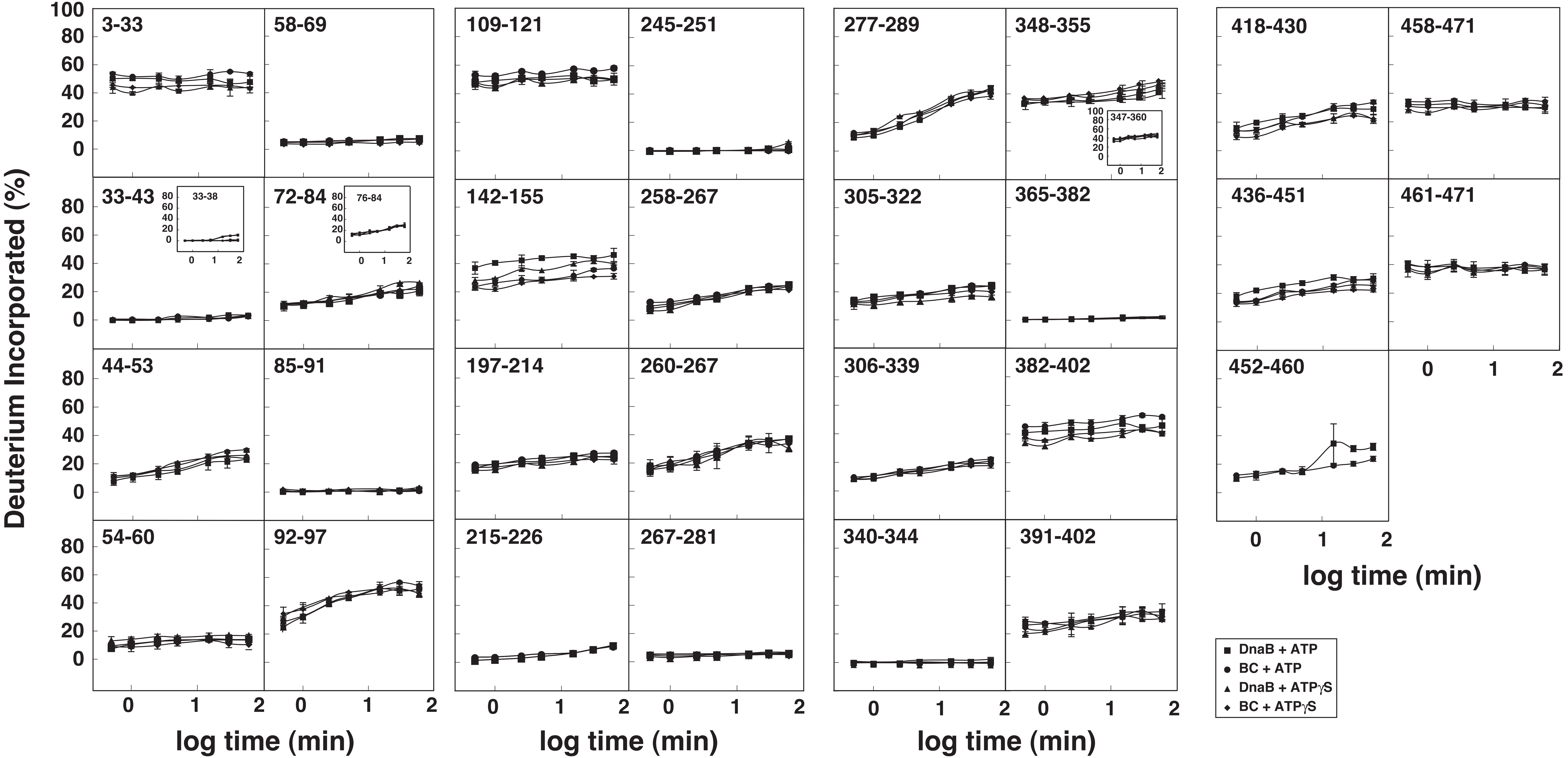
Deuterium Incorporated (%)
log time (min)
3-33
58-69
33-43
33-38
72-84
76-84
44-53
85-91
54-60
92-97
109-121
245-251
142-155
258-267
197-214
260-267
215-226
267-281
277-289
348-355
347-360
305-322
365-382
306-339
382-402
340-344
391-402
418-430
458-471
436-451
461-471
452-460
DnaB + ATP
BC + ATP
DnaB + ATPγS
BC + ATPγS

Figure S4

A Plasmid	Relative Plating Efficiency (42°C/30°C)
Experiment 1	
pET3C	1.8×10^{-3}
pET3C*dnaB*	1.0
pET3C*dnaB*D291A	0.7
pET3C*dnaB*R296A	0.8
pET3C*dnaB*K431A	0.7
pET3C*dnaB*E435A	2×10^{-4}
Experiment 2	
pET3C	1.3×10^{-2}
pET3C*dnaB*	1.0
pET3C*dnaB*W294A	1.5
pET3C*dnaB*I297A	0.7
pET3C*dnaB*T300A	1.1
pET3C*dnaB*M301A	0.6
pET3C*dnaB*I303A	1.0
pET3C*dnaB*L304A	0.3

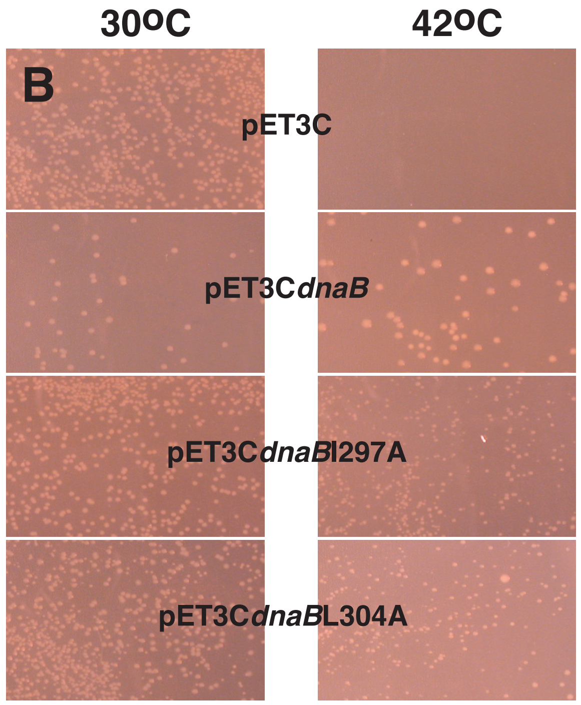

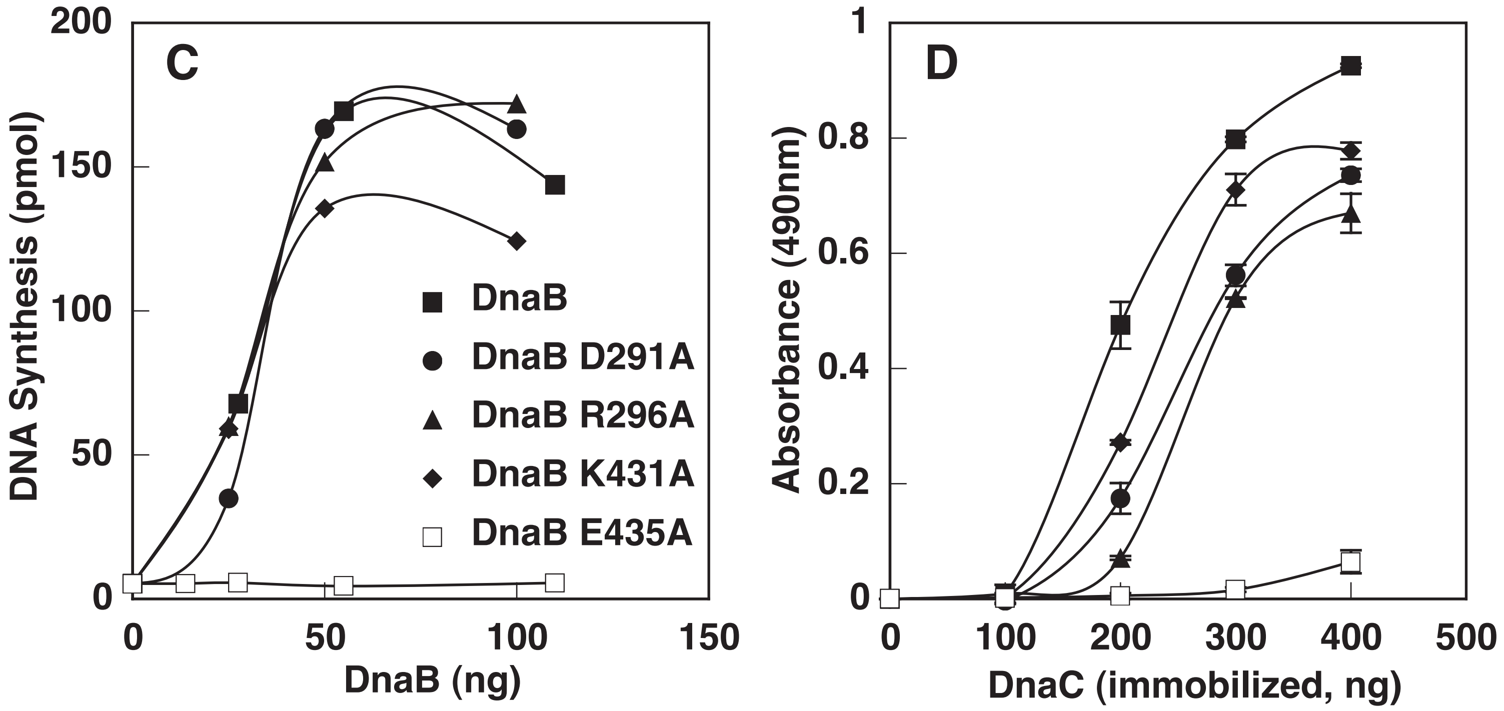

Figure S5

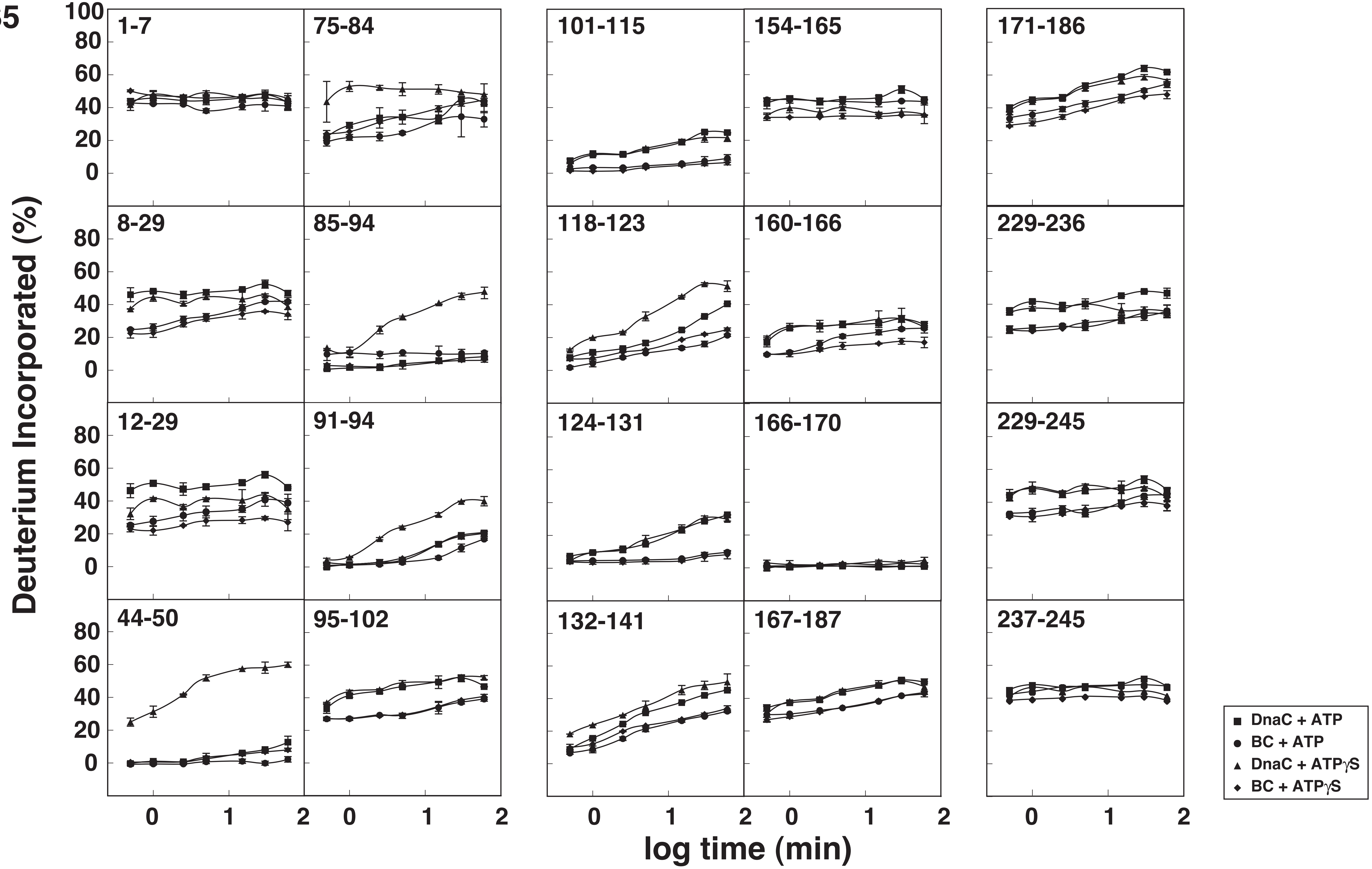

1-7
75-84
101-115
154-165
171-186
8-29
85-94
118-123
160-166
229-236
12-29
91-94
124-131
166-170
229-245
44-50
95-102
132-141
167-187
237-245
Deuterium Incorporated (%)
log time (min)
DnaC + ATP
BC + ATP
DnaC + ATPγS
BC + ATPγS
