# Supplementary figures and images for: DnaC traps DnaB as an open ring and remodels the domain that binds primase
Source: Nucleic Acids Res. 2015 Sep 29;44(1):210–20. doi: 10.1093/nar/gkv961 (PMC4705694; doi:10.1093/nar/gkv961)

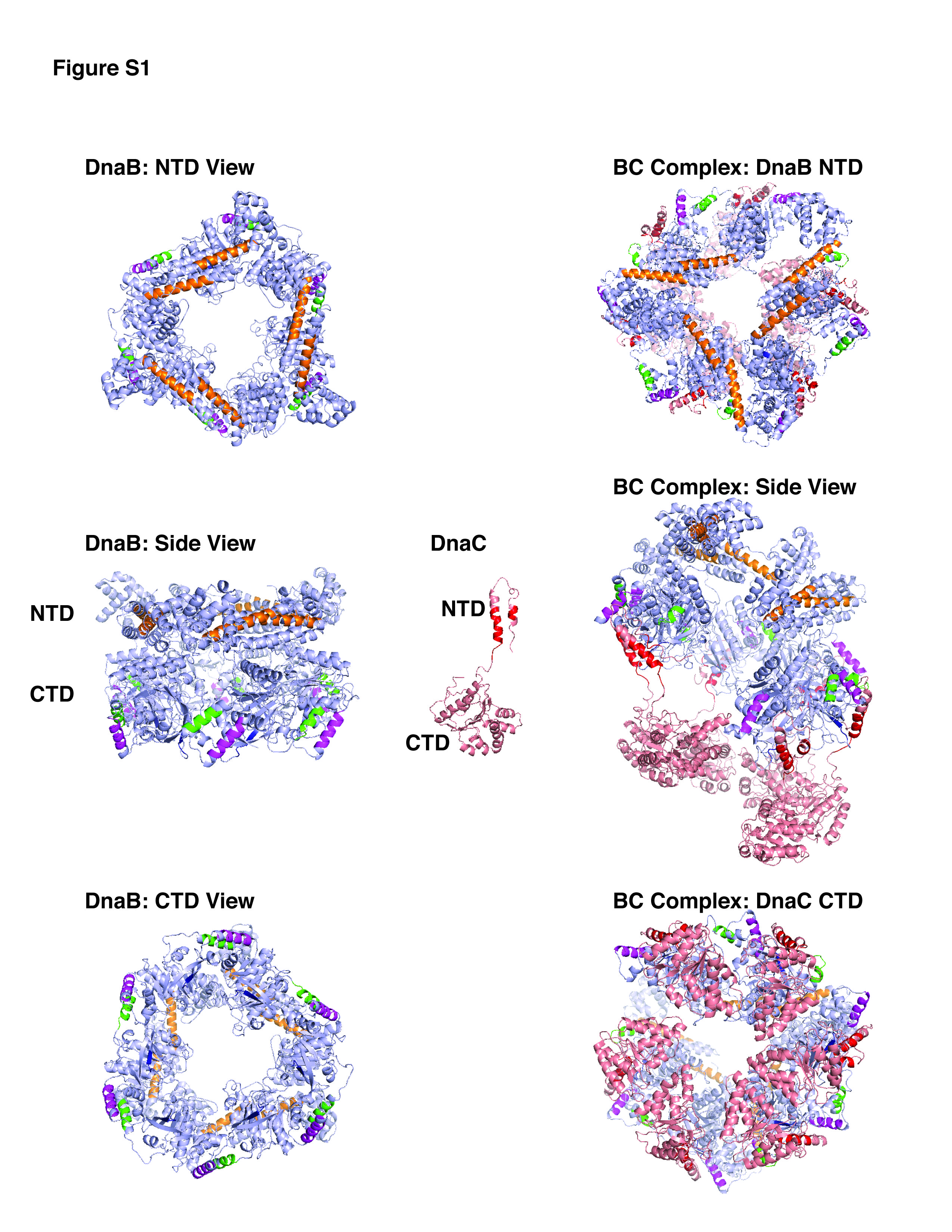

Supplement: SUPPLEMENTARY DATA [file supp_gkv961_nar-01994-m-2015-File008.jpg]

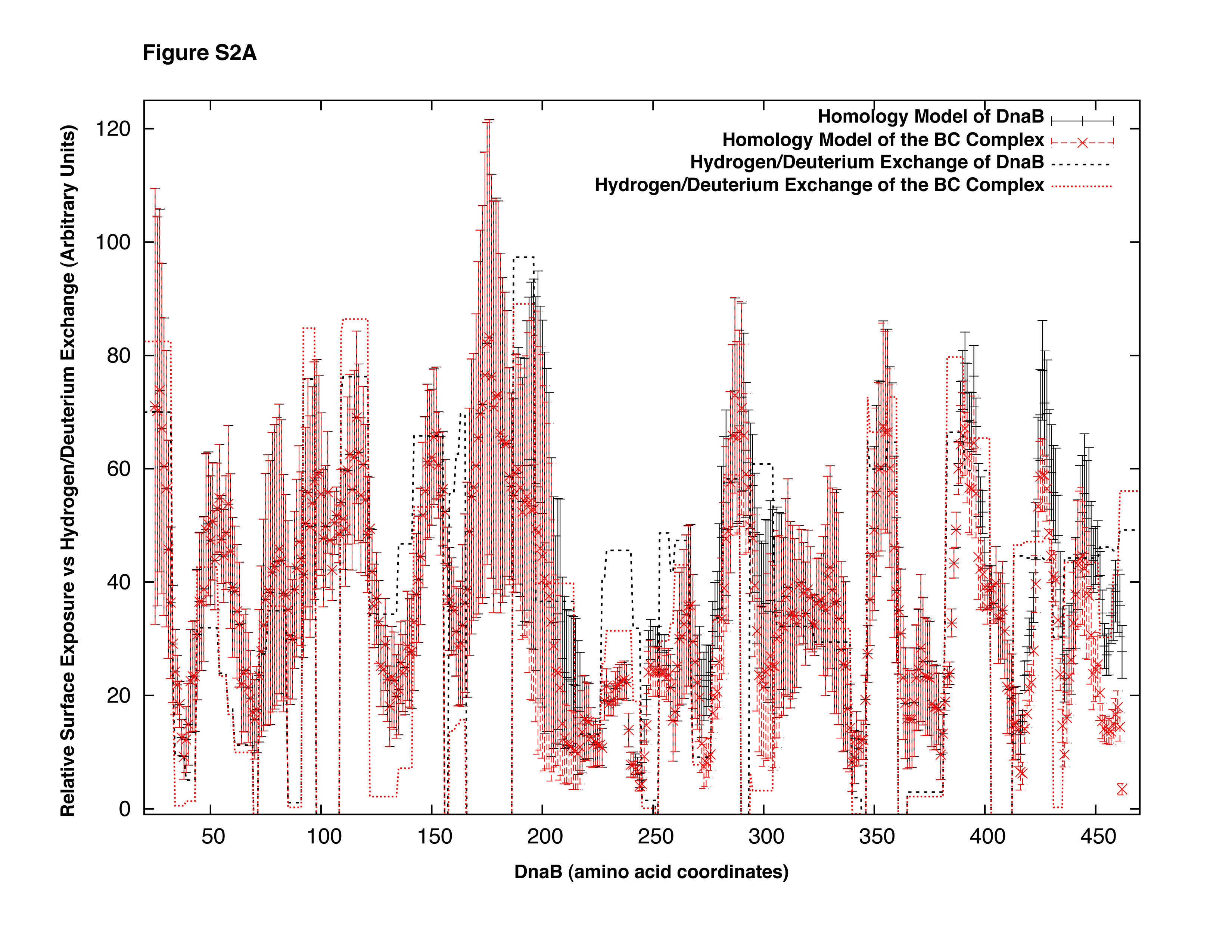

Supplement: SUPPLEMENTARY DATA [file supp_gkv961_nar-01994-m-2015-File009.jpg]

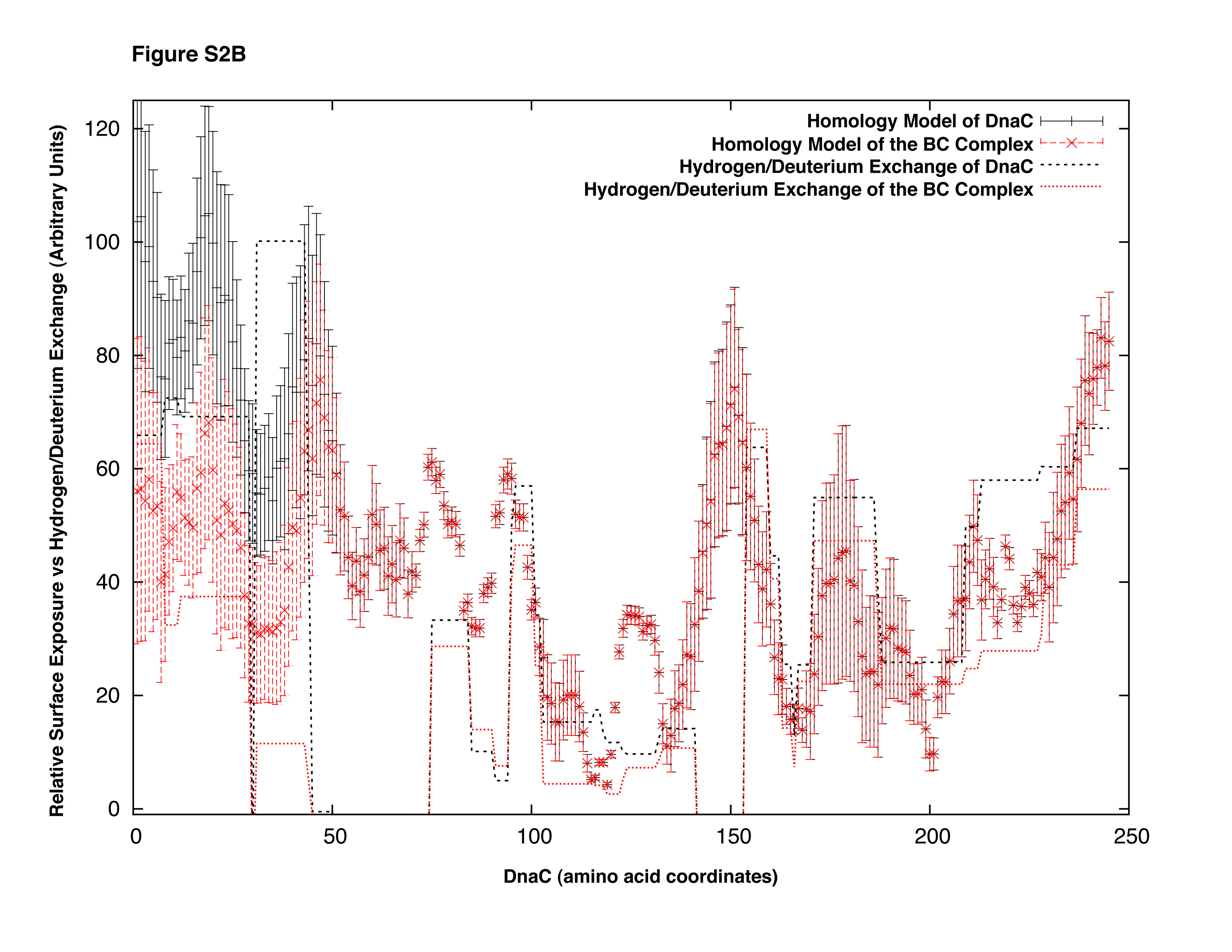

Supplement: SUPPLEMENTARY DATA [file supp_gkv961_nar-01994-m-2015-File010.jpg]

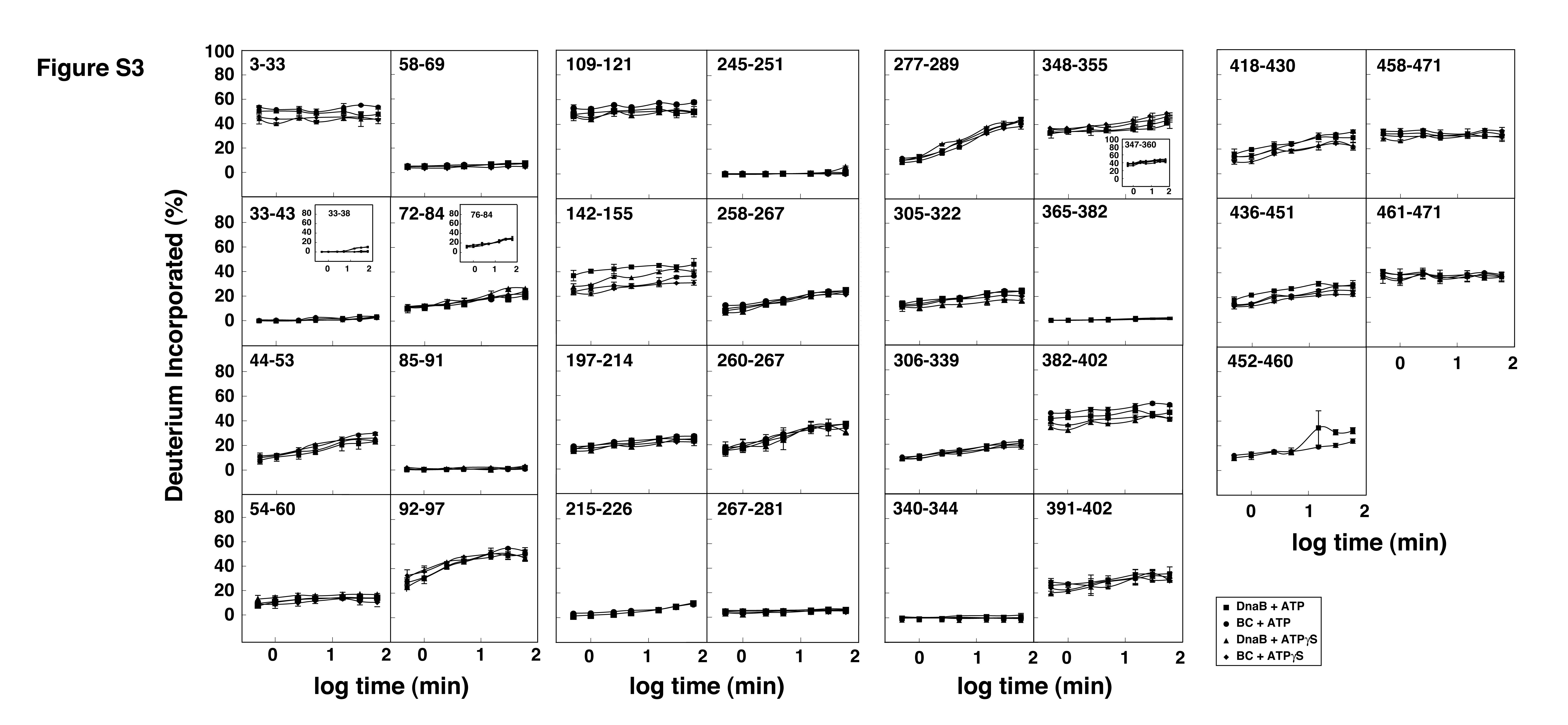

Supplement: SUPPLEMENTARY DATA [file supp_gkv961_nar-01994-m-2015-File011.jpg]

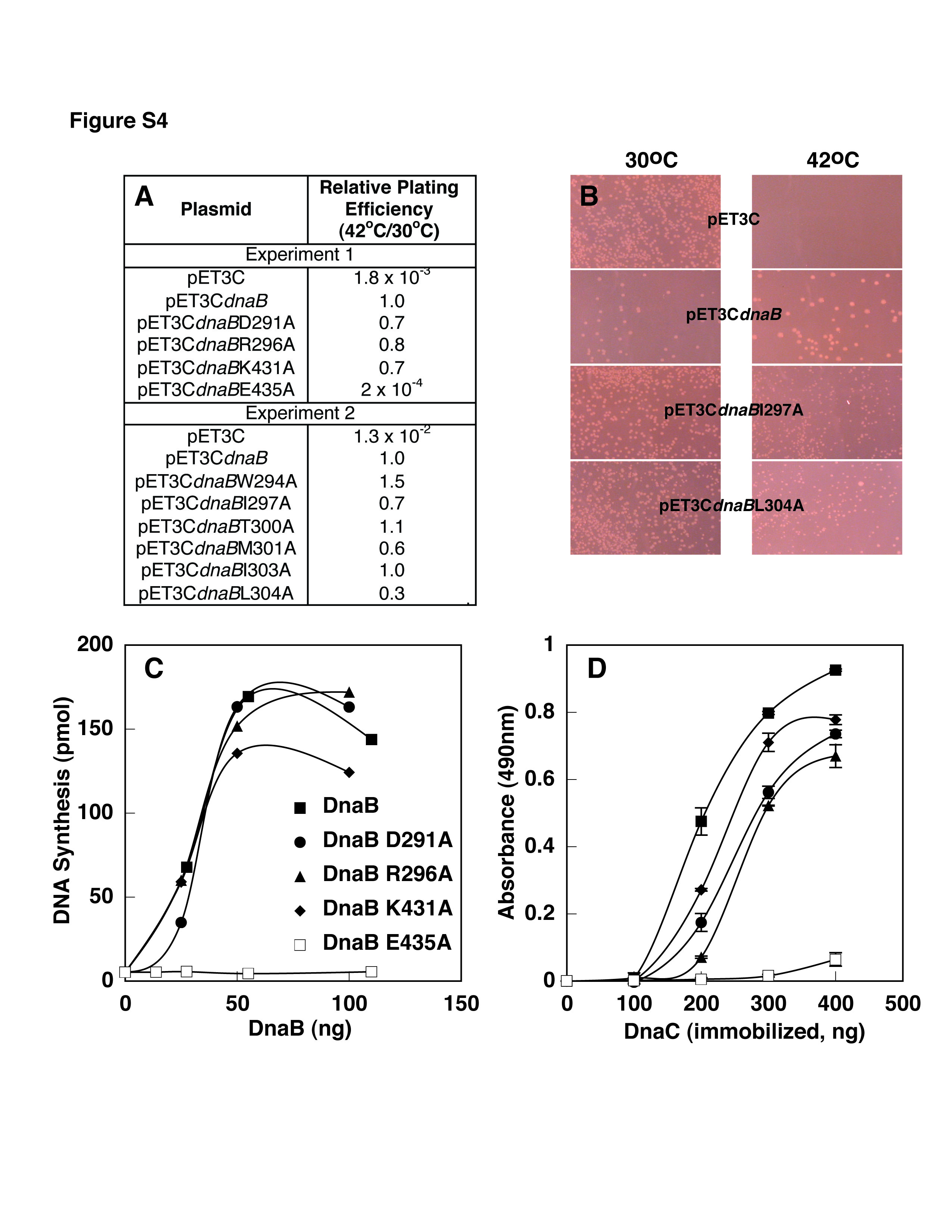

Supplement: SUPPLEMENTARY DATA [file supp_gkv961_nar-01994-m-2015-File012.jpg]

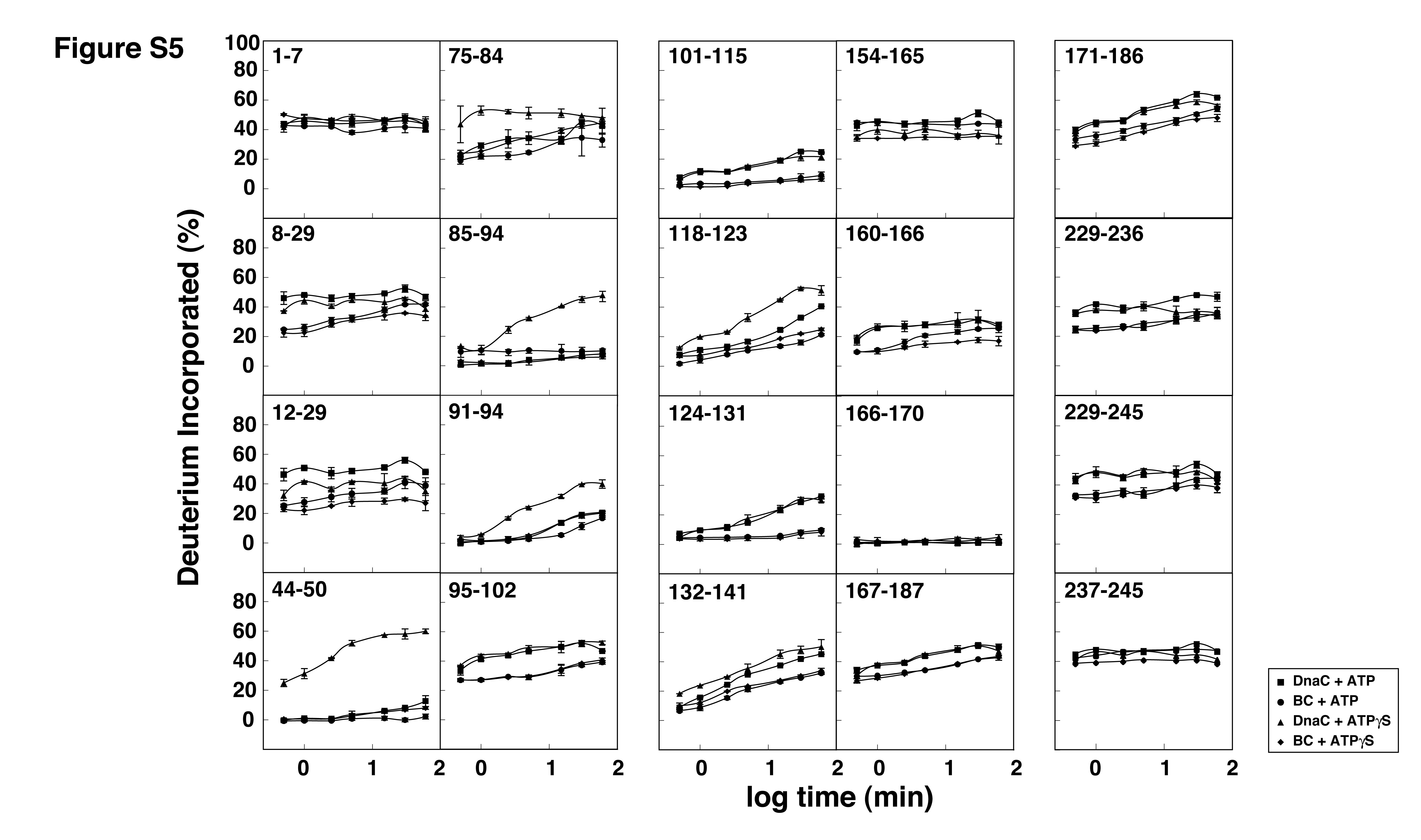

Supplement: SUPPLEMENTARY DATA [file supp_gkv961_nar-01994-m-2015-File013.jpg]
